# Understanding and alleviating informal caregiver burden through the development and validation of a caregiver strain index-based model in Taiwan

**DOI:** 10.1186/s12877-024-05136-5

**Published:** 2024-06-26

**Authors:** Shuo-Chen Chien, Yu-Hung Chang, Chia-Ming Yen, Ying-Erh Chen, Chia-Chun Liu, Yu-Ping Hsiao, Ping-Yen Yang, Hong-Ming Lin, Tsung-En Yang, Xing-Hua Lu, I-Chien Wu, Chih-Cheng Hsu, Hung-Yi Chiou, Ren-Hua Chung

**Affiliations:** 1 Institute of Population Health Sciences, National Health Research Institutes, Miaoli County, 350 Taiwan; 2 National Center for Geriatrics and Welfare Research, National Health Research Institutes, Yunlin County, 632 Taiwan; 3 Graduate Institute of Biomedical Sciences, China Medical University, Taichung City, 404 Taiwan; 4 Department of Risk Management and Insurance, Tamkang University, New Taipei City, 251 Taiwan; 5 School of Public Health, College of Public Health, Taipei Medical University, Taipei, 110 Taiwan

**Keywords:** Informal caregiver burden, Risk factors, Long-term care, Multiple regression analysis, Survival analysis, Behavioral and psychological symptoms of dementia (BPSD)

## Abstract

**Background:**

Quantifying the informal caregiver burden is important for understanding the risk factors associated with caregiver overload and for evaluating the effectiveness of services provided in Long-term Care (LTC).

**Objective:**

This study aimed to develop and validate a Caregiver Strain Index (CSI)-based score for quantifying the informal caregiver burden, while the original dataset did not fully cover evaluation items commonly included in international assessments. Subsequently, we utilized the CSI-based score to pinpoint key caregiver burden risk factors, examine the initial timing of LTC services adoption, and assess the impact of LTC services on reducing caregiver burden.

**Methods:**

The study analyzed over 28,000 LTC cases in Southern Taiwan from August 2019 to December 2022. Through multiple regression analysis, we identified significant risk factors associated with caregiver burden and examined changes in this burden after utilizing various services. Survival analysis was employed to explore the relationship between adopting the first LTC services and varying levels of caregiver burden.

**Results:**

We identified 126 significant risk factors for caregiver burden. The most critical factors included caregiving for other disabled family members or children under the age of three (β = 0.74, *p* < 0.001), the employment status of the caregiver (β = 0.30–0.53, *p* < 0.001), the frailty of the care recipient (β = 0.28–0.31, *p* < 0.001), and the behavioral symptoms of dementia in care recipients (β = 0.28–2.60, *p* < 0.05). Generally, caregivers facing higher burdens sought LTC services earlier, and providing home care services alleviated the caregiver’s burden.

**Conclusion:**

This comprehensive study suggests policy refinements to recognize high-risk caregivers better early and provide timely support to improve the overall well-being of both informal caregivers and care recipients.

**Supplementary Information:**

The online version contains supplementary material available at 10.1186/s12877-024-05136-5.

## Highlights

This study introduced an effective approach for quantifying the burden on informal caregivers, despite the limitations posed by the original dataset’s incomplete coverage of evaluation items typically found in international assessments.

Through analysis of over 28,000 cases, critical risk factors were pinpointed, such as caring for other disabled family members or young children, caregivers’ employment status, care recipients’ frailty, and behavioral symptoms of dementia, emphasizing the need for targeted caregiver support.

Survival analysis revealed that caregivers experiencing higher burdens tend to seek Long-term Care (LTC) services—such as accompaniment on outings, companion services, and home respite services—earlier, which underscores the critical role of these services in supporting caregivers.

Providing home care services alleviates caregiver burden, suggesting policy refinements to enhance the well-being of informal caregivers and care recipients through timely and practical support.

**Supplementary Information:**

The online version contains supplementary material available at 10.1186/s12877-024-05136-5.

## Introduction

Informal caregiving, which involves providing unpaid care to family members or friends through physical assistance, emotional comfort, or rehabilitation, plays a crucial role in global healthcare systems, particularly within the Long-term Care (LTC) fields [1]. Informal caregivers offer essential daily assistance and companionship to individuals facing chronic illnesses, disabilities, or age-related health conditions, often complementing formal care services [2, 3]. The substantial societal impact of informal caregiving is evident, as millions of people engage in this role worldwide [4]. However, the increasing burden on informal caregivers can adversely affect their mental and physical well-being, potentially transforming them from care providers into individuals needing care [5].

Currently, a variety of tools are employed to measure caregiver burden [6], including the Caregiver Strain Index (CSI) [7], Caregiver Burden Inventory (CBI) [8], Zarit Burden Interview (ZBI) [9], Oberst Caregiving Burden Scale (OCBS) [10], and Neuropsychiatric Inventory Caregiver Distress (NPI-D) [11]. These tools are essential for quantifying the informal caregiver burden and further help identify key risk factors that contribute to stress in caregivers. Previous studies have shown that important factors include the care recipient’s level of dependency [12], the caregiver’s relationship to the care recipient, such as a spouse or adult child [13], the duration and intensity of caregiving tasks [13, 14], and characteristics of the caregiver, including age, gender, and mental health status [13, 15]. Identifying these risk factors is crucial for governments and LTC professionals to quickly recognize and support high-burden caregivers, helping to prevent unfortunate incidents and highlighting the importance of identifying these factors in advance. However, the existing LTC datasets may not incorporate these measurement tools, or the included features may not comprehensively cover all necessary evaluation items. Therefore, there is an urgent need for an alternative solution that is not only globally recognized but also adaptable to specific local contexts.

To bridge this gap, our study developed a new tool based on the original CSI, namely CSI-based score, to quantify the burden on informal caregivers. We utilized Taiwanese LTC datasets to explore the feasibility of quantifying caregiver burden when the original dataset lacks certain items from internationally recognized scales. In validating this new suite of this model, we meticulously consider the multifaceted aspects of caregiver burden. We utilized the “Preliminary Screening Indicators for high-burden family caregivers,” which was officially sanctioned by the Taiwanese government, to represent the objective criteria. Additionally, we employ “Expert-assessed”, a subjective evaluation grounded in caregivers’ personal narratives of their experiences. This dual approach enables a more thorough investigation into the indicators’ relevance and efficacy within Taiwan’s LTC landscape.

Following the development and validation of the CSI-based score, we employed this tool to acquire a more nuanced comprehension of the burden experienced by informal caregivers within the Taiwanese LTC framework. First, we used quantitative methods to identify the risk factors for high-burden caregivers and compared them with the existing literature to find key indicators differentiating between high-burden and low-burden caregivers. Second, this study investigated the variations in urgency among caregivers experiencing different levels of burden when seeking LTC services. Finally, we also explored whether utilizing the LTC services leads to substantial improvements in the caregiving burden. Through this research, we hope to provide an empirical basis for policy formulation and resource allocation by depicting a more comprehensive understanding of the fields within LTC.

## Materials and methods

### Assessment and service utilization process in Taiwan’s LTC 2.0 program

Figure S1 illustrates the initial assessment process, utilization of LTC 2.0 services, and subsequent reassessment of the LTC cases (i.e., the care recipient) in Taiwan. When the case or their caregiver applies for LTC resources, a case manager from the local county-level government is dispatched to the case’s home for an interview and evaluation based on the Case Management Evaluation Form (CMEF). The primary caregivers are usually accompanied by a side to answer the survey questions. The CMEF consists of more than 300 questions, including basic information about the case and caregiver, assessments of communication ability, short-term memory, Activities of Daily Living (ADL), Instrumental Activities of Daily Living (IADL), disease history, nutrition, living environment, social activities, emotion and behavior patterns of the case, and care burden, work status, and family support of the caregiver. Along with the CMEF, the case manager also documents the interview summary, typically including information on health conditions, caregiving situation, living environment, and socio-economic status. The case manager constructs a service plan tailored to the case’s personal situation and assesses the degree of need, as indicated by the Case-mix System (CMS) level, which spans from 1 to 8 (with a higher score indicating a higher level of disability). The CMS level, calculated based on the CMEF, determines the maximum subsidy granted by the government. Upon approval of the application, the case can utilize LTC services, such as homecare, professional care, transportation, and respite. A reassessment typically occurs between 300 and 400 days later, following the same procedure and content as the initial assessment.

We analyzed the LTC dataset from Ping-Tung County in Southern Taiwan between August 2019 and December 2022. This dataset comprises basic information about the care recipients of LTC 2.0, questionnaires from the CMEF, and their records of using LTC 2.0 services. The dataset also contains the Preliminary Screening Indicator (PSI), established and issued by the Ministry of Health and Welfare (MOHW) in Taiwan, for identifying high burden family caregivers of LTC recipients. This indicator comprised ten questions (listed in Table S1) and was officially announced on May 10, 2021. Consequently, only data collected after January 2022 contain PSI information. The study was approved by the Institutional Review Board of National Health Research Institutes (Protocol code EC1091216-1 and approval date: November 23, 2021).

### Data preprocessing and distribution of CSI-based score

**Fig. 1:**
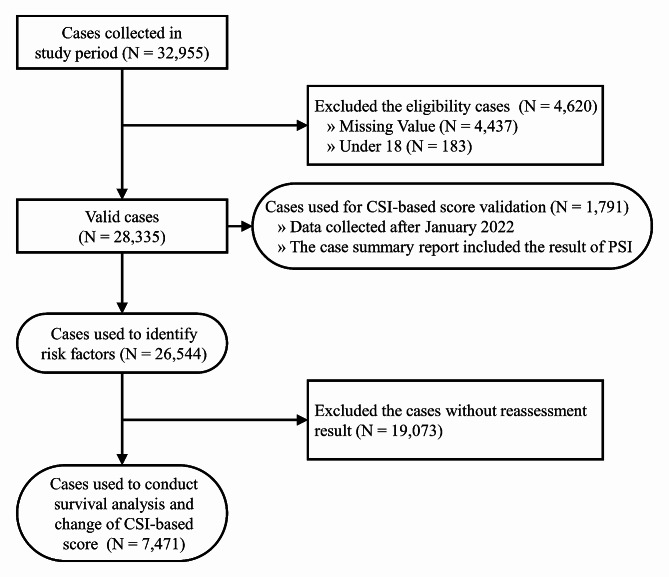
Selection process illustrated in CONSORT diagram

Between August 2019 and December 2022, our study amassed a total of 32,955 cases. Initially, all cases eligible for LTC services were included in our study. However, we excluded cases where either the caregiver or the care recipient was under the age of 18. Figure 1 illustrates the cohort processing steps, depicted through a Consolidated Standards of Reporting Trials (CONSORT) diagram, and highlights the number of excluded cases.

From the initial pool, 4,620 cases were excluded for the following reasons: (1) lack of required values needed to calculate the CSI-based score (*N* = 4,437); and (2) participants under the age of 18 (*N* = 183). Subsequently, we divided the remaining data into two sets (Table S3). The first set, containing 1,791 cases, was utilized to validate the CSI-based score. This validation was conducted using the PSI to assess discriminative power. It included cases with summary reports featuring PSI results, collected after January 2022. The second set was employed to identify informal caregiver burden risk factors using multiple regression analysis. For the survival analysis and to monitor changes in the CSI-based score, we excluded cases lacking a reassessment, resulting in 7,471 cases eligible for further investigation.

### Determining the high-risk cases of informal caregiver burden

Since our dataset lacked international caregiver burden scoring methods and did not cover all evaluation items from these methods, we sought to use an alternative approach closely aligned with globally recognized standards, utilizing our existing LTC dataset. Initially, we investigated commonly used informal caregiver burden measurement indexes such as CSI, ZBI, and BAS to select the index that demonstrated the highest overlapping questionnaires from our CMEF dataset. Upon analysis, we identified the CSI as the index with the most overlap. The CSI is a well-established method for assessing caregiver burden [7]. It consists of 13 items, with responses scored as either 0 or 1. In our study, we identified corresponding questions and calculated CMEF scores to represent the CSI items. Ultimately, we determined that nine items could be explicitly matched (Table 1). To distinguish our adapted version from the original CSI, we referred to the metrics used in our study as CSI-based scores. The detailed validation process of the CSI-based score, employing preliminary screening indicators and expert assessments, is documented in the Supplementary Materials section titled ‘**Using Preliminary Screening Indicators and Expert-assessed to evaluate the CSI-based score.**’

**Table 1:** CSI-based score questions corresponding to the original CSI items

	Items of original CSI	Items of CSI-based score	Scoring criteria
1.	It is confining	The duration that care recipient can be left alone at home (in 24 h).	Over 9 h: +0; 6 h to less than 9 h: +0.2; 3 h to less than 6 h: +0.4; 1 h to less than 3 h: +0.6; Less than 1 h: +0.8; Can not be alone: +1
2.	It is inconvenient	X	
3.	It is a physical strain	Physical burden	Yes: +1; No: +0
4.	Sleep is disturbed	Sleep disturbances	Yes: +1; No: +0
5.	There have been family adjustments	Self-rating for the quality of life.	Very bad: +1; Bad: +0.8; Fair: +0.6; Good: +0.4; Very good: +0.2; Excellent: +0
6.	There have been changes in personal plans	X	
7.	There have been other demands on my time	Need to allocate time to care for other family members	Yes: +1; No: +0
8.	There have been emotional adjustments	X	
9.	Some behavior is upsetting	Distress from care recipient’s behavior	Yes: +1; No: +0
10.	It is upsetting to find the care recipient has changed so much from his/her former self	Care recipient has emotional & behavioral issues (primary caregiver’s perspective).	Has issue(s): +1; No issues: +0
11.	There have been working adjustments	Work affected by caregiving?	Yes: +1; No: +0
12.	It is a financial strain	X	
13.	Feeling completely overwhelmed	Unable to cope with caregiving stress.	Yes: +1; No: +0

Given that caregiver burden is a multifaceted concept, we propose using two methods: PSI and Expert-assessed indicators as distinct evaluation metrics to validate the performance of CSI-based scores, thereby achieving a more comprehensive understanding. The PSI is announced by the Taiwanese MOHW. It identifies caregivers at high risk of burden, utilizing ten criteria ranging from emotional disturbances in care recipients to health issues among caregivers, aiming to refer them for support systematically (Table S1). The Expert-assessed indicator provides a detailed understanding of caregivers’ emotional and psychological challenges through their self-reported experiences, such as “feeling exhausted or fatigued” and “having significant caregiver burden”, highlighting the complexity and depth of caregiving burdens (Table S2). The final status of each case was assigned based on a majority rule from the evaluations of three field experts, requiring at least two agreeing votes. Finally, we evaluated the CSI-based score to predict the classification outcomes involving PSI and Expert-assessed care burden, using multiple performance metrics including accuracy, precision, sensitivity (recall), specificity, F1-score, the area under the receiver operating characteristic (AUROC) curve, and the area under the precision-recall curve (AUPRC) [16–18].

### Impacts of risk factors and LTC services on informal caregiver burden

We carried out three distinct analyses to investigate the interrelations among risk factors, usage of LTC services, and the burden on informal caregivers. Initially, we employed multiple linear regression analysis to identify the risk factors contributing to the burden experienced by informal caregivers. Subsequently, survival analysis was utilized to examine if there was a notable time variation in adopting the first LTC service between caregivers experiencing high burdens and those not. Lastly, through another round of multiple linear regression analysis, we observed the shifts in caregiver burden following the utilization of varying numbers of LTC services. The dataset utilized in this study, sourced from the CMEF, encompasses over 300 features, comprehensively covering aspects relevant to caregivers and care recipients, including the care recipient’s condition, capabilities, and environment, addressing both physical and mental health dimensions.

The first objective aimed to assess the risk factors associated with the burden on informal caregivers, while we utilized multiple linear regression analysis on the variables extracted from the CMEF [19]. The multiple regression model was structured as follows: *CSI-based score (Dependent Variable Y) ~ Features (Independent Variable X) + Gender of the care recipient (Covariate) + Age of the care recipient (Covariate) + Possession of a disability certificate by the care recipient (Covariate) + Presence of dementia in the care recipient (Covariate) + Employing a foreign caregiver (Covariate) + CMS level (Covariate) + Relationship between the caregiver and care recipient (Covariate) + Age of caregiver (Covariate)*.

The second objective aimed to assess whether there is a notable time variation in adopting the first LTC service between informal caregivers in the high-burden group and those not through survival analysis. In this analysis, the “observation window” spanned from the initial assessment to the reassessment. The “event” referred to the case’s first utilization of a specific LTC service. The categorization into the high burden and not-high burden groups of informal caregivers was achieved by identifying the Best Cut-off Point (BCP) of the AUROC curve via the Youden index method. The covariates, which could potentially influence the burden on informal caregivers, were consistent with those used in the multiple linear regression analysis that included risk factors. Subsequently, we utilized the Kaplan-Meier survival curves to depict the time until the event [20]. Meanwhile, a Cox proportional hazards model was used to estimate the hazard ratios for the first use of the top ten most frequently utilized LTC services across various levels of caregiver burden while also adjusting using the same covariates [21].

The third objective was to assess, through another iteration of multiple linear regression analysis, the impact on informal caregiver burden following the use of various numbers of LTC services. Only services utilized by at least 30 unique cases were included in our analysis. These services were categorized into four groups: Category I: Homecare services, Category II: Professional services, Category III: Transportation services, Category IV: Respite services, and Other services. The formula for the multiple linear regression analysis was akin to the one utilized in evaluating risk factors, albeit with a variation in the dependent and independent variables. Specifically, the dependent variable Y was the change in CSI-based score (the reassessment minus the initial assessment), and the independent variable X was the number of specific LTC services utilized by the case. The comprehensive multiple regression model was articulated as: *Change in CSI-based score (Dependent Variable Y) ~ Number of specific LTC services utilized by the case (Independent Variable X) + Gender of the care recipient (Covariate) + Age of the care recipient (Covariate) + Possession of a disability certificate by the care recipient (Covariate) + Presence of dementia in the care recipient (Covariate) + Employing a foreign caregiver (Covariate) + CMS level (Covariate) + Relationship between the caregiver and care recipient (Covariate) + Age of caregiver (Covariate)*.

## Results

### The discrimination ability of CSI-based score

Figure S2 visually represents the classification outcomes as determined by PSI and Expert-assessed. Examples of four different types of cases are provided in Table S4. Based on PSI, 12% of the cases (*N* = 224) were identified as having a high burden, while the remaining 88% (*N* = 1,568) were classified as not exhibiting a high burden. On the other hand, based on the Expert-assessed burden status, 25% of the cases (*N* = 450) were determined to have a high burden, while the remaining 75% (*N* = 1,342) were classified as not exhibiting a high burden.

Interestingly, when comparing these two evaluation metrics, it is important to note that up to 81% (9% + 72%, *N* = 1,456) of cases showed consistent results between PSI and Expert-assessed, with a Cohen’s Kappa statistic of 0.402 (95% CI = 0.352 to 0.451) suggested the slightly moderate agreement, which highlights the concordance between two methods in our analysis despite the differing classification rates (Expert-assessed-1: Expert-assessed-0 vs. PSI-1: PSI-0 = 25:75 vs. 12:88). It reveals that while the PSI metric adopts a stricter evaluation criterion, identifying approximately one in ten as high-burden caregivers, the expert-assessed method categorizes one in four as such.

**Fig. 2:**
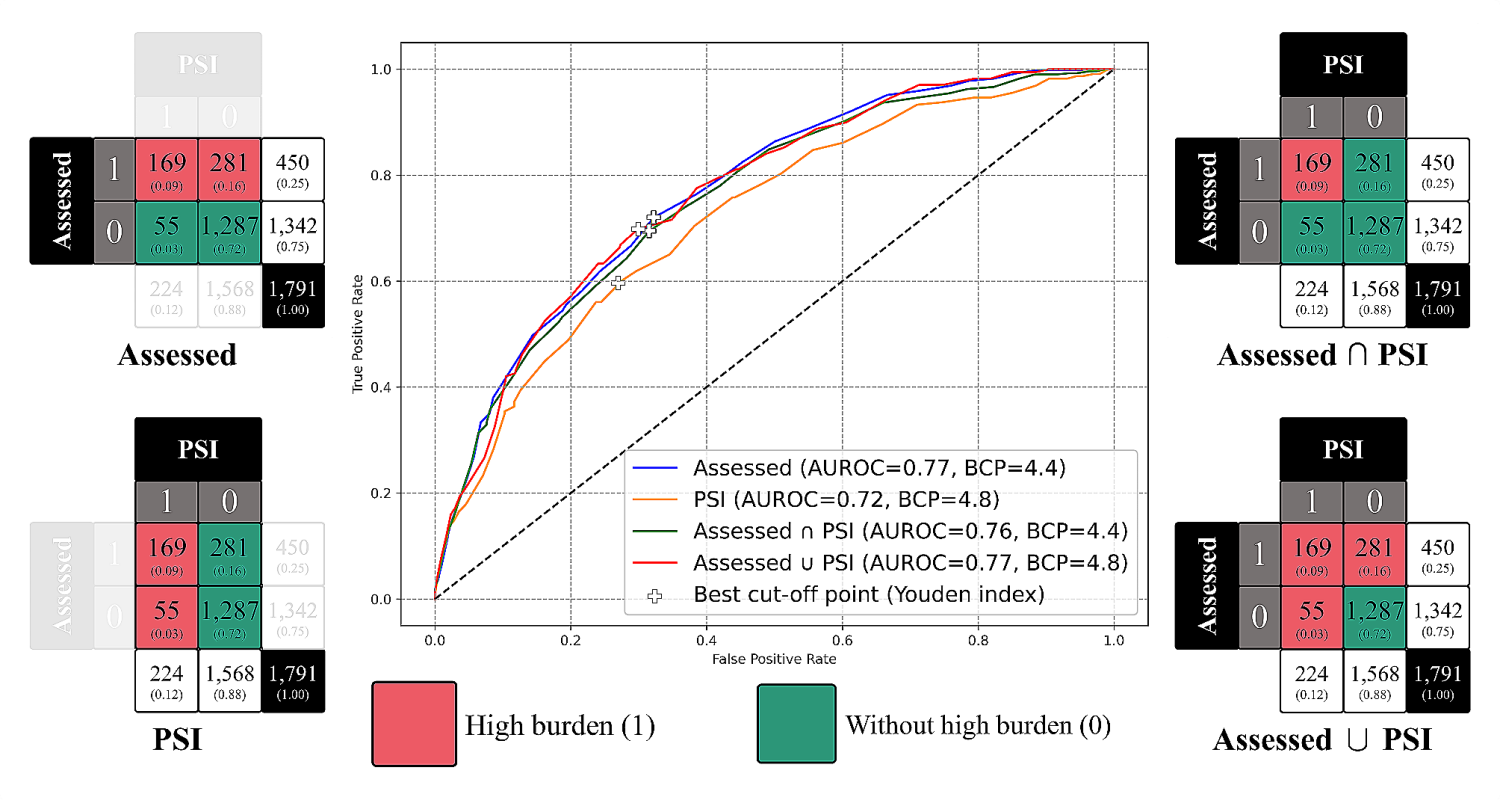
ROC curve for the prediction power of CSI-based score

To assess the discriminatory power of the CSI-based score, we employed it to predict classification outcomes as determined by PSI and Expert-assessed, with results illustrated in the ROC curve shown in Fig. 2. The highest performance was an AUROC of 0.77, achieved in predictions that involved both Expert-assessed categories and their combination with PSI. Considering the CSI-based score is compiled from responses provided by informal caregivers, it naturally follows that predictions related to the Expert-assessed category would exhibit higher accuracy. Furthermore, predictions using solely PSI or the intersection of Expert-assessed and PSI demonstrated AUROC values of 0.72 and 0.76, respectively.

The comprehensive analysis of four models, encompassing detailed metrics such as AUROC, AUPRC, accuracy, precision, recall, specificity, and F1-Score, is shown in Table S5. The optimal threshold for each model was established using Youden’s Index. Focusing on the AUROC, the values for the four models vary slightly, ranging from 0.72 to 0.77. However, when examining the F1-Score, it becomes evident that the Expert-assessed ∩ PSI model outperforms the others with a score of 0.53, markedly surpassing the PSI and Expert-assessed ∪ PSI models, which score 0.34 and 0.31, respectively. Therefore, considering these two critical evaluation metrics, the BCP of the CSI-based score is selected at 4.4, effectively distinguishing between high-burden and not high-burden groups.

Figure S3 displays the distribution of CSI-based scores across the entire dataset (*N* = 28,335), the multiple regression analysis subset (*N* = 26,544), and the validation subset (*N* = 1,791). Within the validation subset, the mean CSI-based score was 4.03, with a standard deviation of 1.7, a minimum of 0.4, and a maximum of 9.0. The scores’ first, second (median), and third quartiles were 2.8, 4.0, and 5.2, respectively. For the entire dataset, the mean score was 4.21, with a standard deviation of 1.71, and the scores ranged from 0.0 to 9.0, with quartiles at 3.0, 4.2, and 5.4, respectively.

### Top ten most significant risk factors for informal caregiver burden

We conducted a multiple regression analysis to examine a total of 303 features in an effort to identify risk factors for informal caregiver burden (Table S6). Comprehensive results can be found in the supplementary material (Fig. S4 - S17). Among the 126 features identified as statistically significant (*p* < 0.05), nearly three-quarters (*N* = 91) demonstrated positive coefficients, in contrast to 35, which showed negative coefficients in their relationship with the CSI-based score. Particularly, the behavioral and psychological symptoms of dementia (BPSD)-related features exhibited high coefficients compared to other binary features, such as “BPSD - Verbal aggression (freq.)” (β = 2.60, *p* = 2.96E-02), “BPSD - Object destruction” (β = 2.58, *p* = 2.21E-03), and “BPSD - Delusions (freq.)” (β = 2.58, *p* = 2.21E-03).

**Fig. 3:**
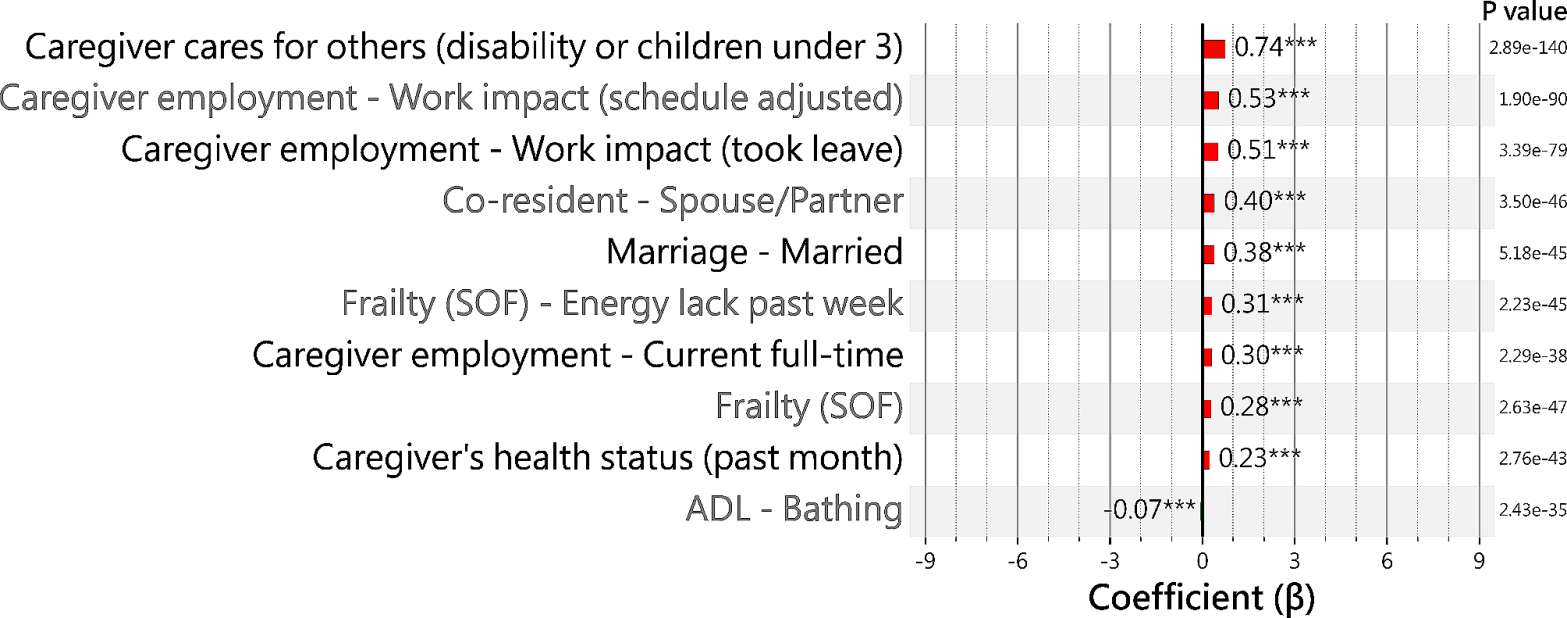
Top 10 minimum p-value features

Figure 3 categorizes the significant risk factors contributing to caregiver burden into attributes related to either caregivers or care recipients. Among caregivers, the most substantial burden is associated with those caring for family members with disabilities or children under three years old, evidenced by a coefficient (β) of 0.74 and a near-zero p-value. The burden is further compounded by work-related factors, such as the necessity to adjust work schedules (β = 0.53, *p* = 1.90E-90) and taking leave from work (β = 0.51, *p* = 3.39E-79). Additionally, the caregiver’s recent health status (β = 0.23, *p* = 2.76E-43) and their employment status, with full-time work inversely related to burden (β = 0.30, *p* = 2.29E-38), are significant. Marital status, specifically being married, is also shown to contribute positively to the caregiver burden (β = 0.38, *p* = 5.18E-45).

Regarding care recipients, the factors affecting caregiver burden include the recipients’ level of frailty, particularly a lack of energy in the past week (β = 0.31, *p* = 2.23E-45), which increases the burden on caregivers. In contrast, the care recipient’s ability to independently perform basic activities, like bathing (β = -0.07, *p* = 2.43E-35), is negatively correlated with caregiver burden, suggesting that recipient self-sufficiency reduces the strain on caregivers. This delineation highlights the distinct yet interconnected factors that influence the degree of strain experienced by caregivers, with each category—caregiver and care recipient—bringing its own set of challenges and mitigating elements to the overall caregiving dynamic.

### The relationship between different burden levels and the first LTC services utilization

This section specifically focuses on the initial engagement with LTC services to investigate whether caregivers experiencing high levels of burden adopt LTC services more promptly than those with lower burdens. The ‘first’ in this context refers to the earliest recorded instance of service utilization following the onset of caregiving responsibilities. This analysis is crucial as it highlights the immediate responses and decisions made by caregivers when confronted with escalating care demands. Survival analysis is employed to distinctly capture the timing of this first adoption among the ten most frequently utilized services, correlating it with the intensity of caregiver burden measured by the CSI-based score threshold of 4.4, as established in Sect. 3.2. Informal caregivers registering a CSI-based score above 4.4 were identified as experiencing a high burden (indicated by a blue line), whereas those with scores below this threshold were considered to have a lower burden (indicated by an orange line). Among the ten services examined, three LTC services—namely, accompaniment to outings, companion services, and home respite services—demonstrated statistical significance. These findings are illustrated in the Kaplan-Meier analysis presented in Fig. S18.

Informal caregivers experiencing a high level of burden are more likely to engage in LTC services. This is evidenced by a Hazard Ratio (HR) of 1.065 for engaging in accompanied outings and 1.116 for utilizing companion services, both of which demonstrate a statistically significant increase (*p* < 0.001) in service usage as the caregivers’ burden intensifies. Similarly, the employment of home respite services is associated with a higher HR of 1.114 (*p* < 0.001), indicating that for every unit increase in the CSI-based score, the probability of using home respite services increases by 1.114 times.

### The impact of LTC services usage on informal caregiver burden

Unlike Sect. 3.3, this section explores the ongoing impact of LTC services on the burden of informal caregivers over an extended period. This part moves beyond the initial adoption to examine how the continued use of various LTC services affects caregiver stress and workload over time. Utilizing multiple linear regression, this analysis evaluates the changes in the CSI-based score from the initial assessment to the reassessment. This approach provides a deeper understanding of the cumulative effects of sustained LTC service utilization, specifically focusing on services engaged by at least 30 unique cases.

We discovered significant insights regarding five specific services (Table 2), with their number of cases (NoC). The service of “Turning over and patting the back” (NoC = 153, β = -2.31E-03, *p* = 0.045), which includes back tapping or trembling for at least 15 min, and “Assistance in performing auxiliary medical procedures” (NoC = 254, β = -4.38E-03, *p* = 0.013), covering tasks such as easing bowel movements with glycerin balls, medication dispensing, blood sugar checks, simple wound care, tube cleaning, and oral suction, were associated with a decrease in caregiver burden. Additionally, “Daytime care services (full-day)” (NoC = 545, β = -2.78E-03, *p* = 0.012), offering comprehensive care from life care to cultural and leisure activities, and “Nutritional meal service” (NoC = 442, β = -1.36E-03, *p* = 0.004), aiding those unable to dine out and supplementing meal preparation for homebound individuals, also showed a negative association with caregiver burden, indicating a reduction in the caregivers’ stress and workload.

**Table 2:** Results from significant MRA on LTC services

Service name and its belonging categories	NoC	Coefficient	*p*
**Category I: Homecare services**			
Turning over and patting the back	153	-2.31E-03^*^	0.045
Assistance in performing auxiliary medical procedures	254	-4.38E-03^*^	0.013
Patrol services	99	1.58E-03^*^	0.032
Daytime care services (full-day)	545	-2.78E-03^*^	0.012
**Other services**			
Nutritional meal	442	-1.36E-03^**^	0.004

Abbreviation: NoC = Number of cases; ^*^ = *p* < 0.05; ^**^ = *p* < 0.01

In contrast, “Patrol services” (NoC = 99, β = 1.58E-03, *p* = 0.032), which entails home visits from 6 AM to 6 PM to check on LTC recipients’ needs and provide basic assistance. The positive association with informal caregiver burden suggests a unique challenge: individuals utilizing this service may often be absent from home, likely due to employment commitments. Previous analyses corroborate that caregivers who are employed and managing caregiving responsibilities simultaneously tend to report higher levels of burden. These findings underscore the nuanced needs of care and the pivotal role of targeted services in supporting the physical and emotional well-being of both patients and their informal caregivers, emphasizing the necessity for a strategic approach in the design and implementation of LTC services that genuinely alleviate the burdens faced by caregivers.

## Discussion

### Main findings

In this study, we developed a CSI-based scoring method as an alternative to quantifying the burden on informal caregivers when the original dataset lacks comprehensive coverage of international evaluation items. By using the CSI-based score, our analysis revealed critical insights under Taiwan’s LTC 2.0 policy, identifying 126 significant risk factors for caregiver burden, notably including responsibilities for other disabled family members or children under 3, the caregiver’s employment status, the care recipient’s frailty, and the presence of BPSD. Additionally, survival analysis showed that informal caregivers facing higher burdens are more likely to engage with LTC services sooner. Furthermore, regarding the LTC services provided by the Taiwanese LTC 2.0 policy, home care services generally alleviate the caregiver burden. However, in contrast, patrol services slightly increased the burden. To the best of our knowledge, this is the first comprehensive analysis that underscores the complex interplay of factors contributing to the informal caregiver burden and explores the relationship between LTC services and burdens.

### Implications

The caregiver burden is a complex phenomenon that varies with the caregiver’s physical and mental state [3, 22]. In validating the CSI-based score, we not only considered the government-issued PSI but also included Expert-assessed based on informal caregivers’ self-reported feelings to define high-burden situations [23, 24]. It’s noteworthy that these Expert-assessed, which directly reflect caregivers’ subjective perceptions of their condition, identified twice as many high-burden cases as the PSI assessments. This discrepancy reveals the stringent criteria of PSI in setting thresholds, aimed at preventing an overload of government LTC resources and ensuring that resources are efficiently allocated to those caregivers most in need, thus reducing system pressure and manpower demands. Conversely, the CSI-based scores developed from our study provide a more adaptable method for identifying high-burden cases, enabling the application of multiple threshold levels to facilitate a more detailed and nuanced identification process. Moreover, benefiting from the CSI-based score, our research has identified a range of factors that significantly increase the burden shouldered by informal caregivers. These insights are pivotal in guiding policy-making efforts, particularly in terms of identifying priority groups for assistance. High-burden informal caregivers are more likely to use LTC services at an earlier stage, indicating that LTC providers could focus on planning and delivering services that specifically address the needs of these caregivers. Indeed, it has been observed that several LTC services can effectively reduce caregiver burden.

Based on our discoveries, we outline a set of proactive recommendations. Firstly, since suicidal intent and domestic violence are high-risk factors of heavy informal caregiver burden, but often unreported due to social concerns [25]. Focusing on visible indicators like BPSD, employment effects, and multiple caregiving duties would be more effective [26]. Secondly, given that caregivers experiencing high levels of burden are more likely to utilize LTC services, it is advisable for providers of these services to prioritize these individuals. Thirdly, considering that LTC services have been shown to reduce caregiver burden effectively, it is crucial to establish a comprehensive support system, such as respite care, flexible work arrangements, and financial assistance. It is also essential to continuously evaluate and adjust these policies and support services to ensure their effectiveness. Lastly, employing machine learning or deep learning methods to predict caregivers’ burdens, similar to forecasting LTC service usage, is proposed to enhance policy effectiveness and support precision [27].

### Compared with previous studies

Numerous previous studies have focused on developing or refining methods to quantify the burden borne by informal caregivers. For instance, Li K.K. and colleagues introduced the Caregiver Needs and Resources Assessment (CNRA), a tool designed to measure unpaid family caregivers’ needs and resources in Hong Kong [28]. It employs various publicly accessible scoring methods to evaluate the tool’s effectiveness from multiple perspectives. Some notable studies have aimed to identify and extract the most critical elements from existing evaluation tools, such as a short version of ZBI, to enhance efficiency [29, 30]. In contrast, our study creates a customized approach that meets international standards and addresses the specific context of Taiwan, incorporating existing features in our dataset. This study demonstrates how, even in the absence of data from internationally recognized assessment tools, key features can be selected from local datasets to develop a quantification method that meets international standards and adapts to local needs.

Our study highlighted the necessity of comprehensively understanding the various factors contributing to the burden on informal caregivers, contrasting with previous studies that often focused on a narrower range of variables. In alignment with prior research, BPSD played a crucial role in adding to the burden of informal caregivers [31]. Other studies have underscored common factors such as female gender, low educational levels, cohabitation with the care recipient, extensive hours dedicated to caregiving, depression, social isolation, financial strain, and the lack of choice in assuming caregiving responsibilities, many of which align with our findings [13]. However, our research uniquely emphasizes the significance of multiple caregiving roles, caregiver employment, and the activity capability of care recipients [32, 33]. Regarding LTC services and their impact on the burden of informal caregivers, our findings indicate that tasks requiring physical strength and specialized knowledge, when managed by home care services, significantly reduce the burden on informal caregivers [34]. Intriguingly, our observations revealed that the utilization of respite services did not markedly lessen the burden on informal caregivers [35]. Taiwan’s LTC policy specifies that only those who have not hired a foreign caregiver, or have one who is not currently active (e.g., due to leave or having gone missing), are eligible to apply for these services. It’s crucial to recognize that employing a foreign caregiver, who offers round-the-clock care, significantly eases the burden on informal caregivers [36]. Therefore, the possible explanation for our finding could be that respite services, primarily intended to fill the gap left when a foreign caregiver is unable to fulfill their caregiving role, may only prevent the burden from worsening rather than improving the current condition.

### Limitations

This study has several limitations. First, this study lacks a detailed analysis of how income levels influence the utilization of LTC services and their potential impact on the varying levels of informal caregiver burden. Second, the participants in our study were primarily from a single geographic region, which might limit the generalizability of our findings to other cultural or geographic contexts. Third, even though our study examined an extensive range of factors related to caregiver burden, there could be additional unmeasured or unknown factors that contribute to the observed associations.

## Conclusion

This study has demonstrated the development of a CSI-based score to quantify the burden on informal caregivers. Our approach serves as an alternative when the original dataset does not fully cover the evaluation items typically used in international assessments. By utilizing the CSI-based score, we identify key risk factors that contribute to the informal caregiver burden, including the caregiver’s responsibilities toward other disabled family members or children under the age of three, the caregiver’s employment status, the frailty of the care recipient, and the presence of BPSD. Moreover, survival analysis revealed that informal caregivers experiencing higher burdens tend to seek LTC services earlier. Furthermore, within the context of Taiwan’s LTC 2.0 policy, it was observed that home care services generally help in reducing caregiver burden. Our findings suggest that efficiently identifying caregivers under high burden can facilitate the early implementation of targeted measures to alleviate this burden. Such measures could include giving priority to high-burden caregivers for LTC services, establishing comprehensive support systems that incorporate respite care, flexible work arrangements, and financial assistance, and enhancing policy effectiveness through the use of machine learning or deep learning techniques to predict caregiver burden.

### Electronic supplementary material

Below is the link to the electronic supplementary material.

Supplementary Material 1

## Data Availability

The datasets generated and/or analyzed during the current study are not publicly available due to licensing agreements but are available from the corresponding author on reasonable request.

## Abbreviations

ADL: Activities of Daily Living
AUROC: Area Under the Receiver Operating Characteristic curve
AUPRC: Area Under the Precision-Recall Curve
BAS: Burden Assessment Scale
BPSD: Behavioral and Psychological Symptoms of Dementia
CBI: Caregiver Burden Inventory
CNRA: Caregiver Needs and Resources Assessment
CMEF: Case Management Evaluation Form
CMS: Case-mix System
CONSORT: Consolidated Standards of Reporting Trials
CSI: Caregiver Strain Index
HR: Hazard Ratio
IADL: Instrumental Activities of Daily Living
LTC: Long-term Care
MOHW: Ministry of Health and Welfare
NPI-D: Neuropsychiatric Inventory of Caregiver Distress
OCBS: Oberst Caregiving Burden Scale
PSI: Preliminary Screening Indicator
SOF: Study of Osteoporotic Fractures
ZBI: Zarit Burden Interview
