# Supplementary material for: Understanding and alleviating informal caregiver burden through the development and validation of a caregiver strain index-based model in Taiwan
Source: BMC Geriatr. 2024 Jun 26;24:558. doi: 10.1186/s12877-024-05136-5 (PMC11200844; doi:10.1186/s12877-024-05136-5)
Supplement: Supplementary file 1 — Supplementary Material 1 [file 12877_2024_5136_MOESM1_ESM.docx]

Supplementary Materials

Using Preliminary Screening Indicators and Expert-assessed to evaluate the CSI-based score

Caregiver burden is a multifaceted concept, the perception of which can vary significantly among individuals. Some caregivers may subjectively feel overwhelmed by their responsibilities, a sensation that might appear relatively minor to external observers, especially when compared to cases that have faced more severe challenges. To gain a comprehensive understanding of caregiver burden, this study employs two distinct evaluation metrics: Preliminary Screening Indicators (PSI) and Expert-assessed indicators.

**Preliminary Screening Indicator (PSI)**

The PSI, detailed in **Table S1**, originates from the comprehensive guidelines issued by the Taiwanese Ministry of Health and Welfare. This table methodically outlines ten distinct indicators that collectively serve to identify caregivers facing potentially high burdens. These indicators encompass a broad spectrum of challenges, from dealing with care recipients exhibiting severe emotional disturbances to managing the caregiver's own health issues that might impede their caregiving capabilities. For instance, the table includes criteria such as the age and specific situations of the caregivers, such as being elderly or indigenous, which are factors contributing to their classification as high-burden. Other criteria focus on the lack of alternative caregiving options or the caregiver's need to simultaneously care for multiple individuals with long-term care needs. This structured approach enables a systematic assessment, ensuring that caregivers who meet any combination of these criteria receive the necessary support and resources.

**Table S1. Preliminary Screening Indicators for high-burden family caregivers.**

| **No.** | **Indicator** | **Operational Definition** |
| --- | --- | --- |
| 1 | Care recipient with severe emotional disturbances making caregiving challenging | The care recipient exhibits behavior and psychological symptoms (BPSD), self-harm or harming others, aggression, disruption, or unusual behaviors (e.g., wandering, delusions, shouting, making strange noises), causing the caregiver to experience physical and psychological stress, making it difficult to provide care. |
| 2 | Elderly caregivers | 1. Caregiver aged 65 or above.  2. Indigenous caregiver aged 55 or above.  Note: If the caregiver is under 18 years old, priority should be given to notify the care center for care arrangement adjustments and to report to the social safety net system. |
| 3 | No prior caregiving experience | 1. Became a family caregiver due to family changes.  2. Facing changes in the care recipient’s physical condition or disease (e.g., new pressure sores, catheter or BPSD), and the caregiver lacks sufficient caregiving knowledge. |
| 4 | No alternative caregiver | 1. Bears the primary caregiving work for more than 20 hours per week, without assistance from other family members, friends, or caregiving resources.  2. Due to traditional cultural or gender factors, the caregiver or care recipient is reluctant to seek help or resist using resources. |
| 5 | Caregiving for more than two persons | Also responsible for caregiving for 2 or more individuals qualifying for long-term care, with disabilities, holding a developmental delay certificate, or needing to allocate time to care for other family members (e.g., children under 3 years old, psychiatric patients). Note: If identified as a dual elderly family (primary caregiver over 60 years old, disabled individual over 35 years old), or a family with 2 or more individuals with disabilities or psychiatric conditions, should report to the disability or social safety net system. |
| 6 | Caregiver’s health conditions affect ability or willingness to care | 1. The caregiver has mental or other health conditions limiting their caregiving capacity or willingness.  2. Exhibits symptoms like depression, anxiety, sleep disorders, etc., limiting caregiving capacity or willingness.  3. The caregiver holds a disability certificate or has a severe illness card (including cancer) affecting their caregiving capacity or willingness. Note: If suspected of suffering from |
| 7 | Ineligible for government resources, changes in eligibility, or sudden emergency needs | 1. Needs financial assistance but is ineligible due to owning real estate or changes in household registration, etc., not meeting government regulations.  2. Unable to afford long-term care expenses due to sudden incidents. |
| 8 | Changes in caregiving situation within the past 3 months | 1. Caregiver has acute medical needs.  2. Change in the care recipient’s condition (e.g., frequent hospital admissions).  3. Gap periods of foreign caregivers (e.g., missing or changing employers) or other interruptions in caregiving resources. |
| 9 | History of domestic violence or negligence between caregiver and care recipient | 1. Caregiver reports having had violent thoughts or neglect, regardless of official reporting records.  2. Assessed as likely having experienced domestic violence or neglect, regardless of official reporting records. |
| 10 | Caregiver has attempted suicide or harbored suicidal thoughts | 1. Caregiver previously had suicidal thoughts due to caregiving stress, attempted suicide, had a concrete suicide plan, or prepared the means for suicide. 2. Has expressed thoughts of suicide or ending both their own and the care recipient’s life in conversation. |
| Referral Standards for Family Caregiver Support Service Locations: Must meet one of the following conditions:  1. Meets criteria of Indicator 9 or 10  2. Meets any two indicators  3. Other situations assessed by professionals as requiring referral | | |

**Expert-assessed**

The Expert-assessed indicator, as illustrated in **Table S2**, captures the nuanced, personal experiences of caregivers through their self-reported words. This table is a compilation of expressions and terms that caregivers might use when describing their burdens and stresses, providing insights into the emotional and psychological landscape of caregiving. From feeling a general caregiving burden to specific states of high stress, overwhelming burden, and physical fatigue, the table spans a wide array of self-reported feelings and conditions. For example, it includes terms reflecting acute psychological pressures, such as "feeling exhausted or fatigued", and more specific scenarios like "tense caregiving stress" or dealing with "high stress and burden". This qualitative data is crucial for understanding the depth and variety of caregiving experiences, offering a richer, more personal perspective on the challenges faced. It emphasizes the importance of subjective experiences in evaluating the overall burden, acknowledging that the impact of caregiving can extend far beyond observable criteria to include deeply felt emotional and psychological effects.

**Table S2. Examples of terms for Expert-assessed of caregiving burden.**

| **English Translation** | **Chinese Terms** |
| --- | --- |
| Caregiving burden and stress | 照顧(護)負荷/負擔/壓力 |
| High caregiving stress | 照顧壓力大/照顧壓力甚大/照顧壓力繁重/照顧壓力過大 |
| Significant caregiver burden | 照顧負荷高/照顧負荷大 |
| Overwhelming caregiver burden | 照顧負荷(過)重 |
| Unable to bear caregiving stress | 無法承受照顧壓力 |
| Experiencing caregiving stress | 有照顧壓力/備感照顧負荷大 |
| Heavy caregiver burden | 照顧負荷沉重/照顧負荷較重 |
| Caregiver's stress and burden | 照顧者壓力/照顧者壓力負荷 |
| Heavy caregiving stress | 照顧壓力沉重 |
| High stress and burden | 壓力負荷重/壓力負荷高/壓力負荷大 |
| Physical burden | 體力負荷 |
| Psychological stress | 心理有壓力/精神疲累 |
| Heavy stress | 壓力沉重 |
| Feeling exhausted or fatigued | 備感壓力/備感疲憊/深感照顧疲累/身心疲憊 |
| Tense caregiving stress | 照顧壓力緊繃/照顧壓力略高 |

By integrating these two indicators, the study aims to provide a holistic assessment of caregiver burden, facilitating a deeper understanding of both the observable and experiential aspects of caregiving challenges. This approach not only recognizes the diverse nature of caregiver experiences but also enhances the ability to identify those in need of support, thereby informing the development of targeted interventions.

Supplementary Tables

**Table S3. Descriptive statistics of demographics variables.**

|  | | **Total cohort**  **(N = 28,335)** | | | **CSI-based score validation**  **(N = 1,791)** | | | **Multiple regression analysis**  **(N = 26,544)** | | |
| --- | --- | --- | --- | --- | --- | --- | --- | --- | --- | --- |
|  | | Mean (SD) / N | | Null  (%) | Mean (SD) / N | | Null  (%) | Mean (SD) / N | | Null  (%) |
| *Dependent Variable* | | | | | | | | | | |
| **CSI-based score** | |  | 4.20 (1.71) | 0 |  | 4.03 (1.70) |  |  | 4.22 (1.71) | 0 |
| *Independent Variable* | | | | | | | | | | |
| **Recipient's gender** | | 0 | | | 0 | | | 0 | | |
|  | Male |  | 11,910 |  |  | 722 |  |  | 11,188 |  |
|  | Female |  | 16,425 |  |  | 1,069 |  |  | 15,356 |  |
| **Care recipient's age** | | 0 | | | 0 | | | 0 | | |
|  | Age |  | 76.45 (11.9) |  |  | 76.55 (11.84) |  |  | 76.45 (11.9) |  |
| **Caregiver's age** | |  | | 0 |  | | 0 |  | | 0 |
|  | Age |  | 57.74 (13.08) |  |  | 57.33 (12.37) |  |  | 57.77 (13.13) |  |
| **Relationship** | | 0 | | | 0 | | | 0 | | |
|  | Spouse |  | 7,182 |  |  | 406 |  |  | 6,776 |  |
|  | Son |  | 8,117 |  |  | 529 |  |  | 7,588 |  |
|  | Daughter |  | 6,465 |  |  | 437 |  |  | 6,028 |  |
|  | Daughter/Son in law |  | 3,003 |  |  | 182 |  |  | 2,821 |  |
|  | Parents |  | 797 |  |  | 51 |  |  | 746 |  |
|  | Siblings |  | 949 |  |  | 59 |  |  | 890 |  |
|  | Employed caregivers |  | 42 |  |  | 2 |  |  | 40 |  |
|  | Other |  | 1,780 |  |  | 125 |  |  | 1,655 |  |
| **Hiring an employed caregiver** | | <0.001 | | | 0 | | | <0.001 | | |
|  | Yes |  | 2,721 |  |  | 185 |  |  | 2,536 |  |
|  | No |  | 25,613 |  |  | 1,606 |  |  | 24,007 |  |
| **Presence of dementia in the care recipient** | |  |  | 0 |  |  | 0 |  |  | 0 |
|  | Yes |  | 2,567 |  |  | 106 |  |  | 2,461 |  |
|  | No |  | 25,768 |  |  | 1,685 |  |  | 24,083 |  |
| **Possession of a disability certificate by the care recipient** | | 0 | | | 0 | | | 0 | | |
|  | Yes |  | 10,898 |  |  | 660 |  |  | 10,238 |  |
|  | No |  | 17,437 |  |  | 1,131 |  |  | 16,306 |  |
| **Care recipient's CMS level** | | 0 | | | 0 | | | 0 | | |
|  | Level 1 |  | 972 |  |  | 56 |  |  | 916 |  |
|  | Level 2 |  | 4,253 |  |  | 302 |  |  | 3,951 |  |
|  | Level 3 |  | 4,939 |  |  | 369 |  |  | 4,570 |  |
|  | Level 4 |  | 5,508 |  |  | 356 |  |  | 5,152 |  |
|  | Level 5 |  | 4,686 |  |  | 264 |  |  | 4,422 |  |
|  | Level 6 |  | 3,065 |  |  | 186 |  |  | 2,879 |  |
|  | Level 7 |  | 2,568 |  |  | 134 |  |  | 2,434 |  |
|  | Level 8 |  | 2,344 |  |  | 124 |  |  | 2,220 |  |
| **Care recipient's educational level** | | 0 | | | 0 | | | 0 | | |
|  | Illiterate |  | 8,095 |  |  | 441 |  |  | 7,654 |  |
|  | Literate (No formal education) |  | 2,080 |  |  | 178 |  |  | 1,902 |  |
|  | Elementary/Primary education |  | 10,039 |  |  | 626 |  |  | 9,413 |  |
|  | Junior high school |  | 2,992 |  |  | 182 |  |  | 2,810 |  |
|  | Senior/Vocational high school |  | 3,136 |  |  | 235 |  |  | 2,901 |  |
|  | Special education |  | 90 |  |  | 4 |  |  | 86 |  |
|  | Junior college/technical college |  | 485 |  |  | 29 |  |  | 456 |  |
|  | Bachelor's degree |  | 1,124 |  |  | 76 |  |  | 1,048 |  |
|  | Master's degree and above |  | 119 |  |  | 13 |  |  | 106 |  |
|  | Other |  | 175 |  |  | 7 |  |  | 168 |  |
| **Care recipient's ADL** | |  |  | 0 |  |  | 0 |  |  | 0 |
|  | Barthel index |  | 58.44 (26.38) |  |  | 60.57 (24.53) |  |  | 58.3 (26.5) |  |
| **Care recipient's IADL** | |  |  | 0 |  |  | 0 |  |  | 0 |
|  | For male (Five-point total) |  | 1.89 (1.33) |  |  | 2.06 (1.28) |  |  | 1.87 (1.33) |  |
|  | For female (Eight-point total) |  | 3.18 (1.9) |  |  | 3.48 (1.82) |  |  | 3.16 (1.9) |  |
| Abbreviation: SD = Standard deviation; CMS = Case-mix System; ADL = Activities of Daily Living; IADL = Instrumental Activities of Daily Living | | | | | | | | | | |

**Table S4. Examples of case summary reports classified by PSI and Expert-assessed.**

|  | | **Preliminary Screening Indicators (PSI)** | |
| --- | --- | --- | --- |
|  |  | 1 | 0 |
| **Expert-assessed** | 1 | …Caregiver Overburden Issue: The case's wife complains of feeling physically exhausted from taking care of their second son with moderate intellectual disability, especially now that the primary caregiver has been hospitalized for about a month, preventing her from working outside. Respite services are suggested to alleviate caregiving stress….According to the preliminary screening indicators, the case qualifies under criteria 4. no alternative caregiver available, 7. ineligible for government resources, changes in eligibility, or sudden emergency needs, and 10. caregiver has had suicidal attempts or thoughts. Referral to a family caregiver support service location is recommended…. | ...According to the preliminary screening indicators, the case qualifies under criteria 3. no alternative caregiver available and 6. caring for a person with dementia. However, it does not meet the criteria for referral. The care professional and Unit A will continue to provide support....Family Caregiver Services: The case's eldest daughter is experiencing significant caregiving stress. It is recommended that the family apply for services to alleviate this stress. The eldest daughter indicated that she currently has no intention of applying for services.... |
|  | 0 | …The case involves multiple family members taking turns in caregiving. Additionally, the mother currently does not require care. The eldest son was informed about the possibility of referral to home caregiving services, but he indicated no need for such services at this time, as the eldest daughter-in-law and the eldest granddaughter are also assisting with care. With multiple people sharing caregiving responsibilities, there is no request for referral to family caregiver support services….According to the preliminary screening indicators, the case qualifies under criteria 3. lack of prior caregiving experience, 4. no alternative caregiver available, 5. needing to care for more than two individuals, and 8. changes in the caregiving situation, meeting the standards for referral to family caregiver support service locations…. | …The parent-child relationship and interactions among the siblings are harmonious, with good family dynamics and supportive relationships…. According to the preliminary screening indicators, the case qualifies under criteria 4. no alternative caregiver available and 5. need to care for more than two persons, but does not meet the criteria for referral to family caregiver support services…. |

**Table S5. Model performance among different indicators.**

| **Model** | **1** | **0** | **AUROC** | **AUPRC** | **Accuracy** | **Precision** | **Recall** | **Specificity** | **F1-Score** | **BCP of CSI-based score** |
| --- | --- | --- | --- | --- | --- | --- | --- | --- | --- | --- |
| **Expert-assessed** | 463 (0.25) | 1,367  (0.75) | 0.77 | 0.53 | 0.70 | 0.44 | 0.66 | 0.71 | 0.53 | 4.4 |
| **PSI** | 225  (0.12) | 1,605  (0.88) | 0.72 | 0.29 | 0.71 | 0.24 | 0.60 | 0.73 | 0.34 | 4.8 |
| **Expert-assessed ∩ PSI** | 170  (0.09) | 1,660  (0.91) | 0.76 | 0.55 | 0.70 | 0.47 | 0.64 | 0.72 | 0.54 | 4.4 |
| **Expert-assessed ∪ PSI** | 518  (0.28) | 1,312  (0.72) | 0.77 | 0.28 | 0.72 | 0.20 | 0.67 | 0.73 | 0.31 | 4.8 |
| Abbreviation: PSI = Preliminary Screening Indicator; AUROC = Area under the receiver operating characteristic; AUPRC = Area under the precision-recall curve; BCP = Best Cut-off Point  Noted: The best cut-off point was calculated by the Youden Index. | | | | | | | | | | |

**Table S6. Features employed for analysis in our study.**

| **Feature Name** | **Total** | **NoF** | **Type** |
| --- | --- | --- | --- |
| Related to the care recipient | 289 |  |  |
| Care recipient's basic information |  | 23 | C & N |
| Care recipient's disability type |  | 12 | C |
| Care recipient's communication ability |  | 9 | C & N |
| Care recipient's short-term memory ability |  | 5 | C & N |
| Care recipient's ADLs and IADLs scale |  | 21 | C & N |
| Care recipient's pain, skin, and wound condition |  | 22 | C |
| Care recipient's nutrition, joint, and frailty condition |  | 19 | C & N |
| Care recipient's exists disease |  | 73 | C |
| Care recipient's medical assistance demand |  | 22 | C |
| Care recipient's swallowing ability |  | 7 | C |
| Care recipient's dementia training status |  | 5 | C |
| Care recipient's fall history |  | 7 | C |
| Care recipient's co-resident status |  | 14 | C |
| Care recipient's living environment status |  | 19 | C |
| Care recipient's social participation status |  | 10 | C |
| Care recipient's BPSD |  | 21 | C |
| Related to caregiver | 14 |  |  |
| Caregiver's basic information |  | 6 | C & N |
| Caregiver's employment status |  | 8 | C |
| Total Features: 303 | | | |
| Abbreviations: NoF = Number of features; C = Categorical; N = Numerical; ADL = Activities of Daily Living; IADL = Instrumental Activities of Daily Living; BPSD = Behavioral and Psychological Symptoms of Dementia | | | |

**Table S7. The result of the multiple regression analysis for LTC services.**

| **Services** | **N** | **Coefficient** | | ***p*** |
| --- | --- | --- | --- | --- |
| **Category I: Homecare services** |  |  |  |  |
| Basic personal hygiene | 775 | 2.39E-04 |  | 0.744 |
| Basic daily care | 1,077 | 2.93E-04 |  | 0.501 |
| Vital signs measurement | 176 | 7.48E-05 |  | 0.961 |
| Assistance with feeding or tube feeding | 471 | -2.63E-04 |  | 0.603 |
| Meal care | 1,598 | -7.69E-04 |  | 0.079 |
| Assistance with bathing and shampooing | 3,504 | 1.63E-04 |  | 0.633 |
| Turning over and patting the back | 153 | -2.31E-03 | * | 0.045 |
| Stretching limb joint | 621 | 7.03E-05 |  | 0.927 |
| Assistance in going up (down) stairs | 35 | -8.23E-04 |  | 0.761 |
| Accompanying outings | 2,193 | 2.84E-04 |  | 0.172 |
| Accompanying to medical appointments | 591 | -6.63E-04 |  | 0.947 |
| Household assistance | 3,498 | -9.72E-04 |  | 0.073 |
| Shopping, collection, or delivery service | 1,409 | 1.13E-04 |  | 0.725 |
| Assistance in performing auxiliary medical procedures | 254 | -4.38E-03 | * | 0.017 |
| Companion services | 1,559 | 3.45E-04 |  | 0.202 |
| Patrol services | 99 | 1.58E-03 | * | 0.032 |
| Assistance with shampooing | 88 | -1.90E-04 |  | 0.959 |
| Daytime care services (full-day) | 545 | -2.78E-03 | * | 0.012 |
| Daytime care services (half-day) | 203 | 1.65E-03 |  | 0.397 |
| Home-based caregiving services (full-day) | 46 | -3.14E-03 |  | 0.348 |
| Home-based caregiving services (half-day) | 31 | 8.36E-04 |  | 0.866 |
| Community-assisted bathing | 125 | -2.69E-03 |  | 0.327 |
| Community transportation services | 557 | -5.40E-04 |  | 0.331 |
| **Category II: Professional services** |  |  |  |  |
| ADLs rehabilitation care -- at home | 342 | 6.94E-04 |  | 0.966 |
| IADL rehabilitation, ADL rehabilitation care | 330 | 1.43E-02 |  | 0.611 |
| Bedridden or long-term limited mobility care | 98 | -1.85E-02 |  | 0.497 |
| **Category III: Transportation services** |  |  |  |  |
| Transportation and pick-up | 1,176 | 1.69E-04 |  | 0.932 |
| **Category IV: Respite services** |  |  |  |  |
| Home respite services | 1,760 | 4.09E-03 |  | 0.150 |
| Daycare center respite services | 186 | 4.67E-03 |  | 0.269 |
| Institutional residential and small-scale multi-function (night respite) | 117 | 1.24E-02 |  | 0.411 |
| **Other services** |  |  |  |  |
| Nutritional meal | 442 | -1.36E-03 | ** | 0.004 |
| Abbreviation: ADL = Activities of Daily Living; IADL = Instrumental Activities of Daily Living; * = *p* < 0.05; ** = *p* < 0.01; *** = *p* < 0.001.  Noted: Multiple regression analysis equation: *Change in CSI-based score (Dependent Variable Y) ~ Number of specific LTC services utilized by the case (Independent Variable X) + Gender of the care recipient (Covariate) + Age of the care recipient (Covariate) + Possession of a disability certificate by the care recipient (Covariate) + Presence of dementia in the care recipient (Covariate) + Employing a foreign caregiver (Covariate) + CMS level (Covariate) + Relationship between the caregiver and care recipient (Covariate) + Age of caregiver (Covariate).* | | | | |

Supplementary Figures


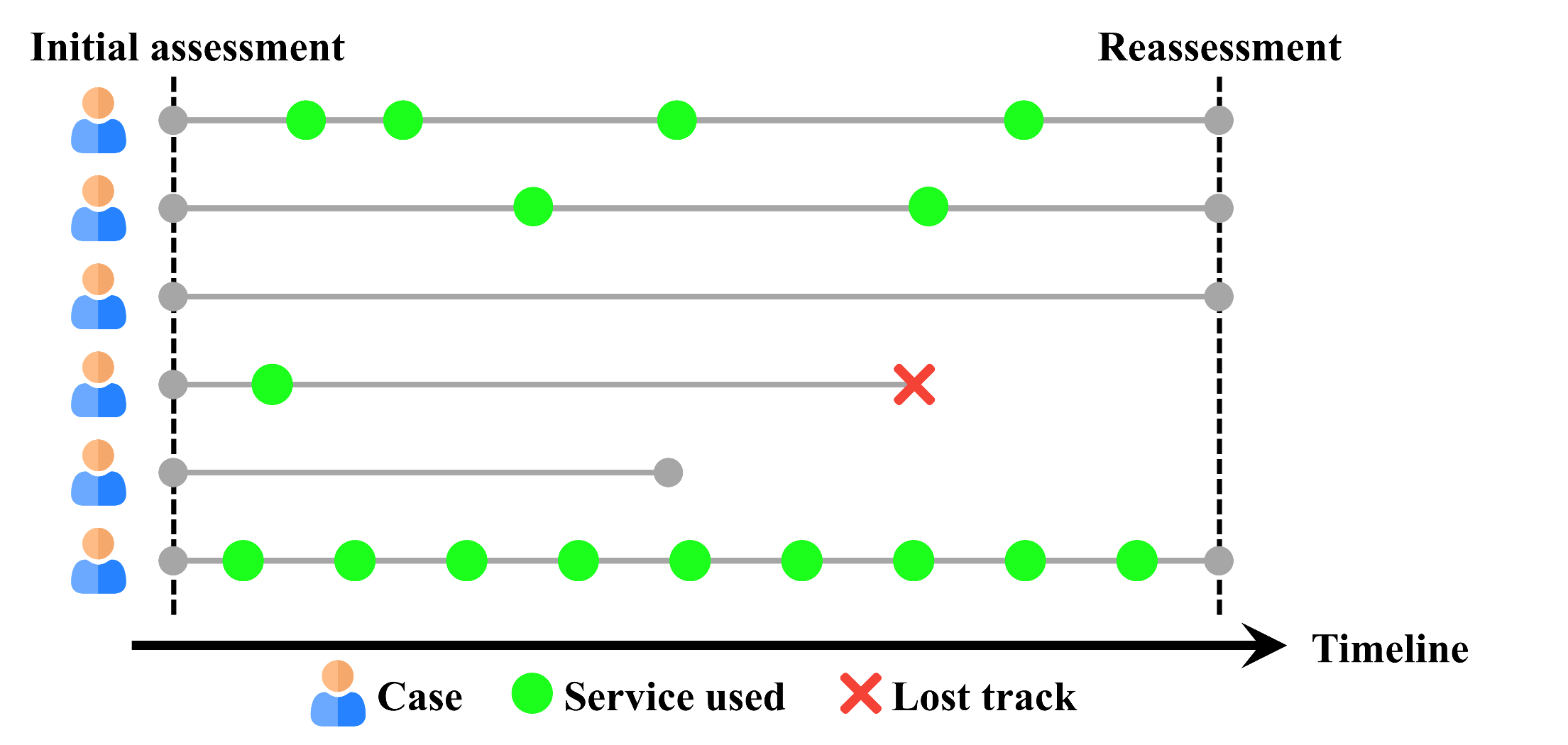


**Fig S1. Schematic diagram illustrating the process of LTC service usage.**

**
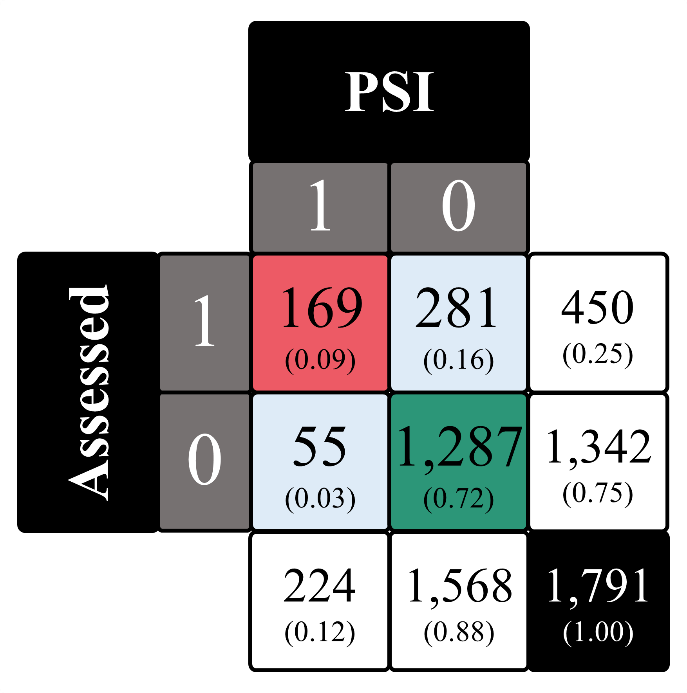
**

**Fig S2. Classification results based on two indicators.**


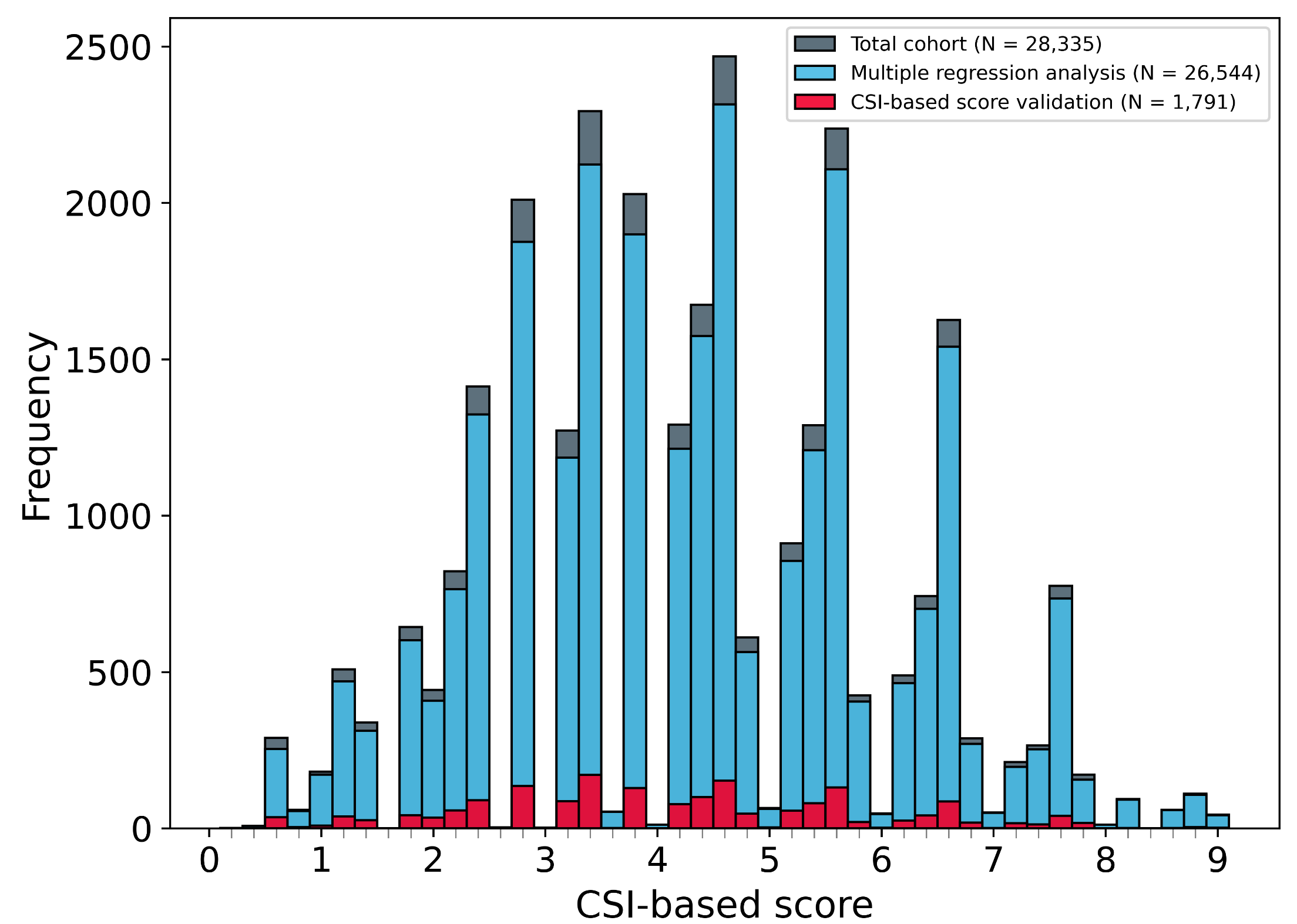


**Fig S3. Distribution of the CSI-based score.**


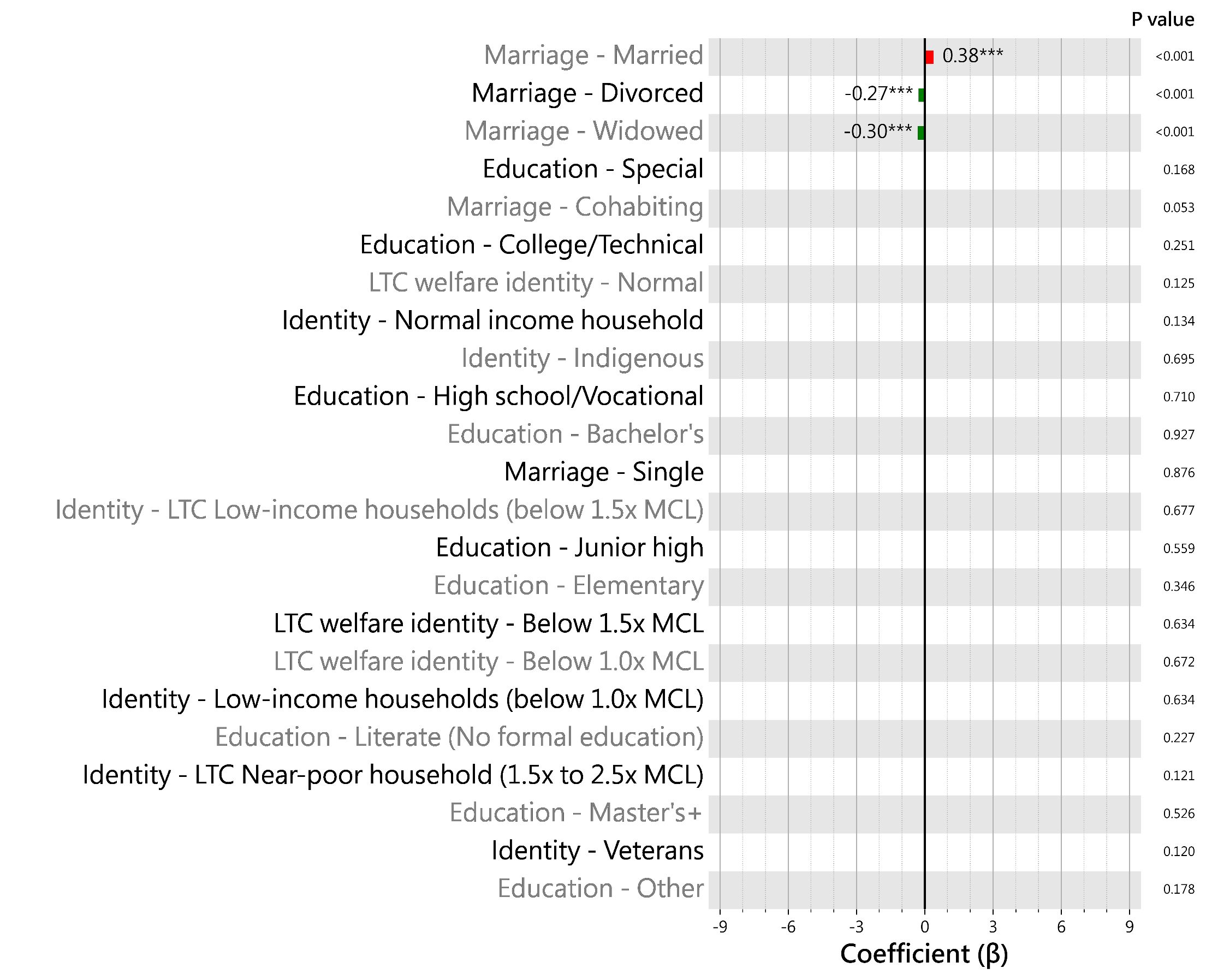


**Fig S4. MRA results- care recipient's identity, marital status, and educational level.**

**
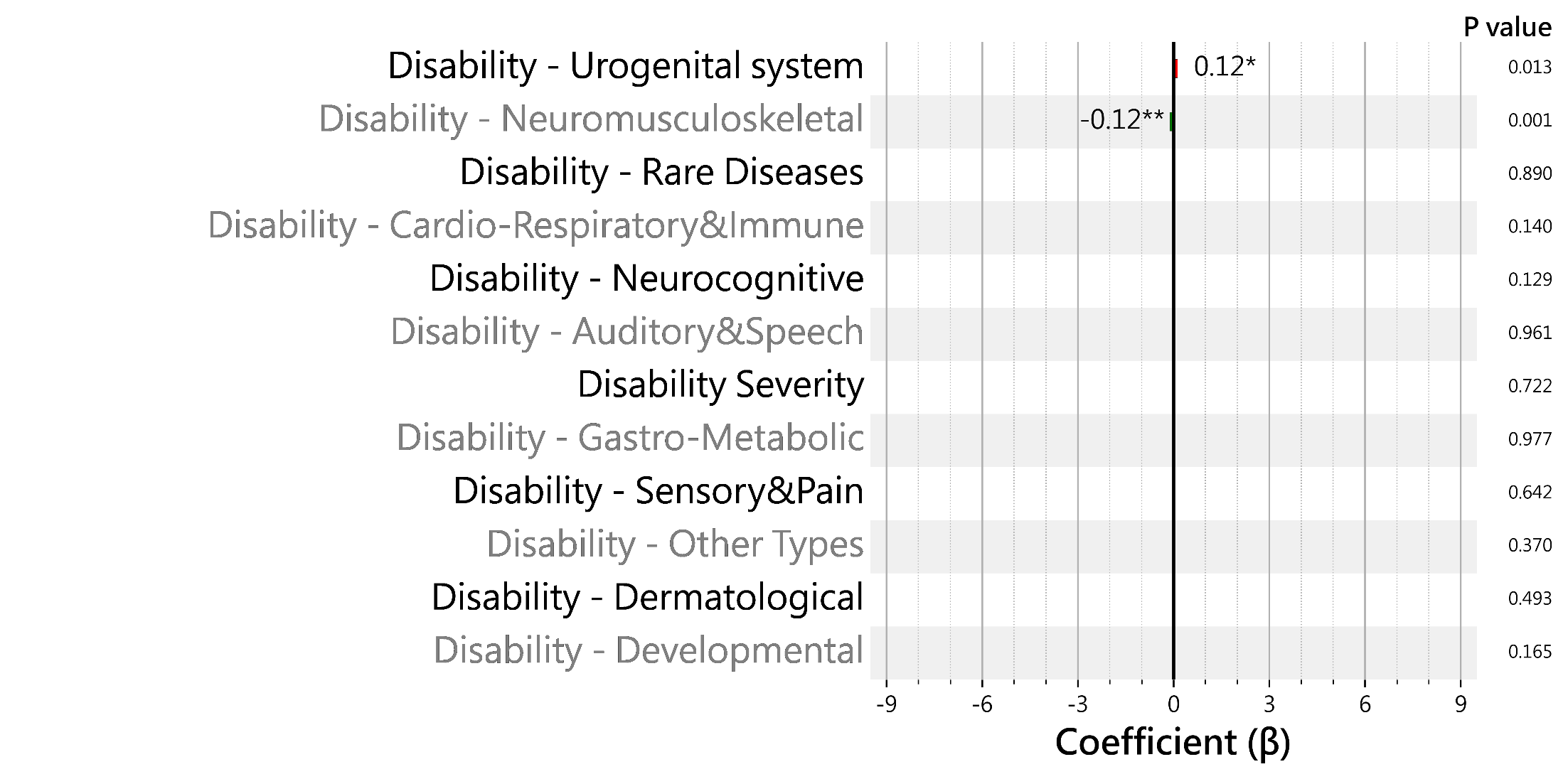
**

**Fig S5. MRA results- care recipient's disability status.**

**
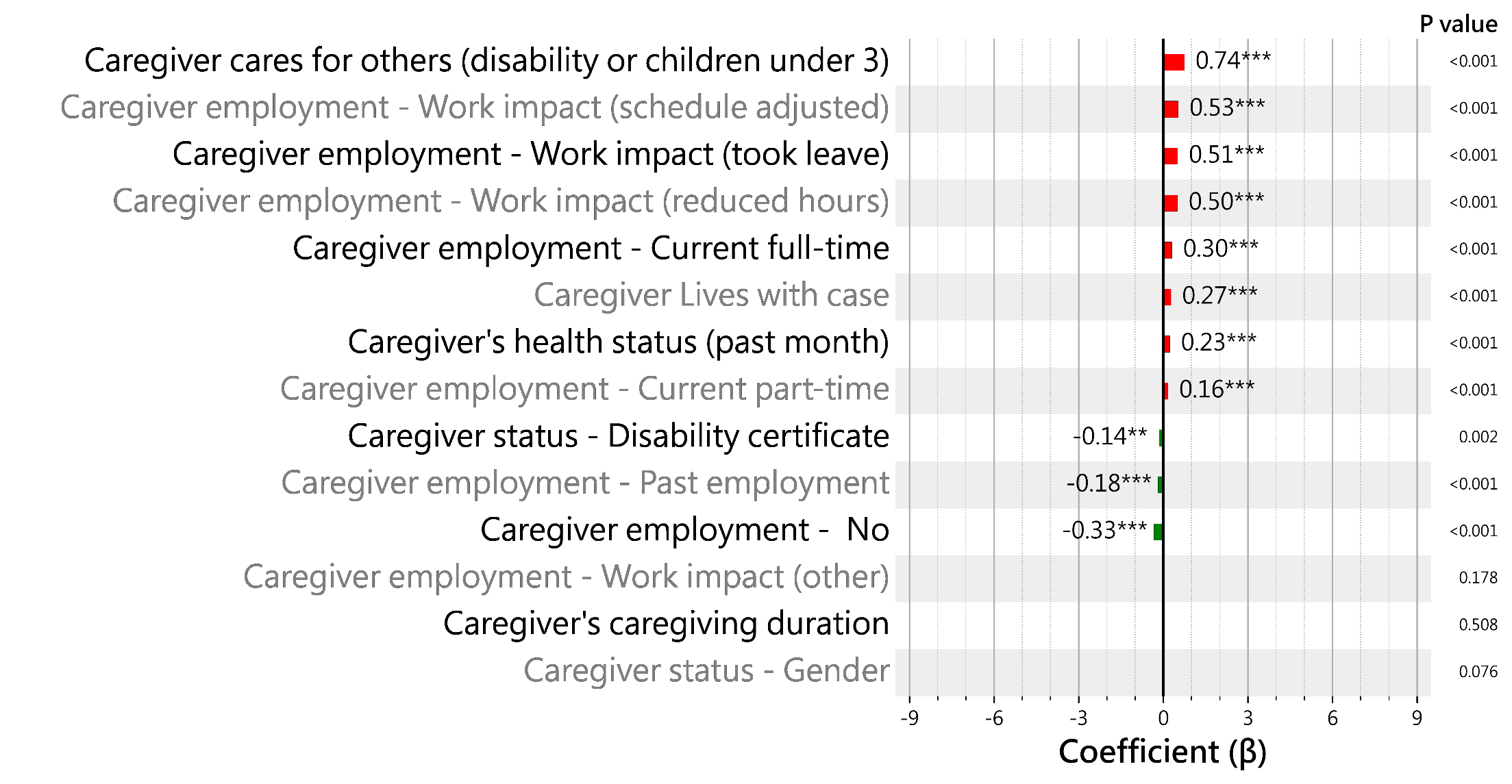
**

**Fig S6. MRA results- caregiver status.**

**
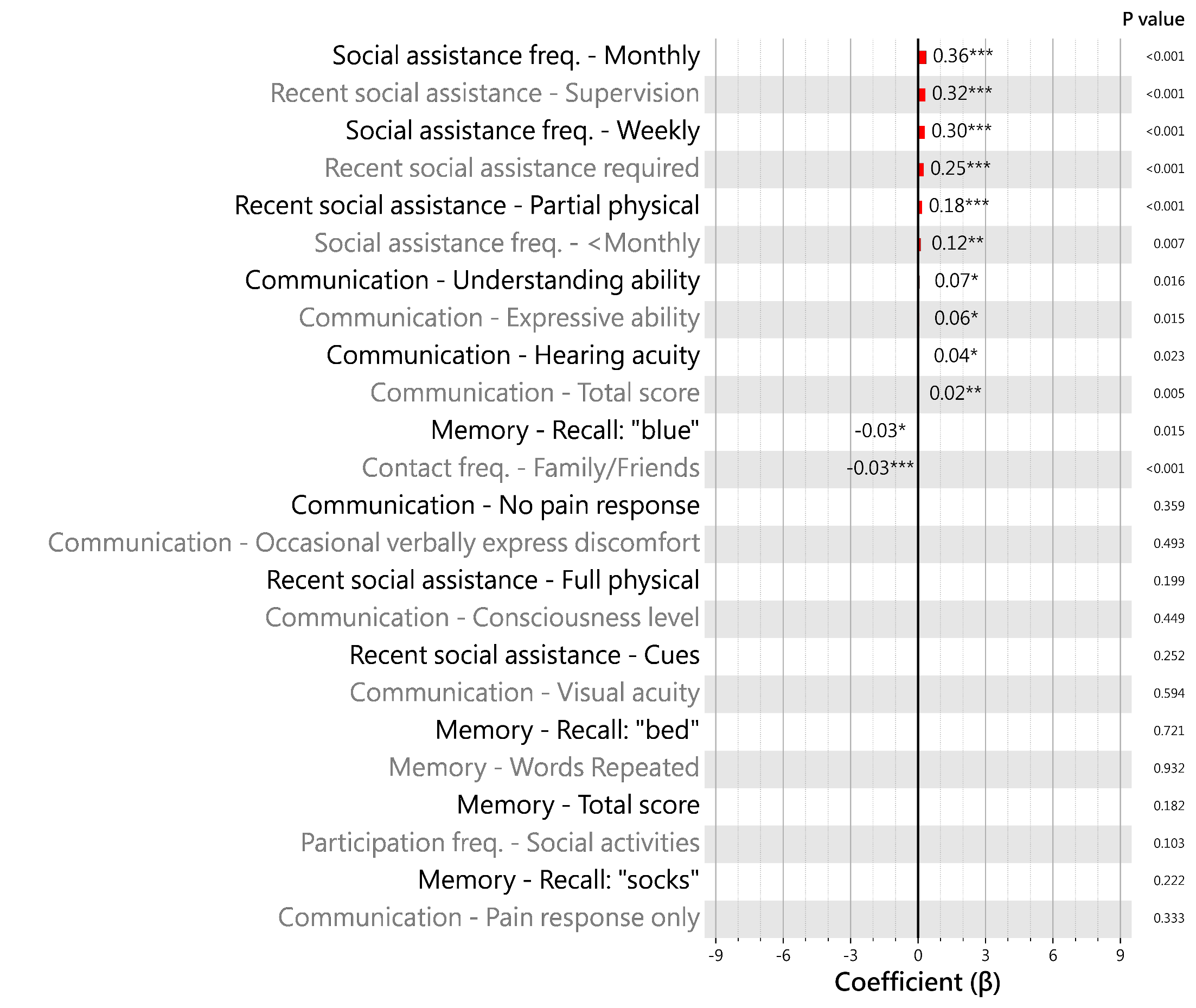
**

**Fig S7. MRA results- care recipient's communication, short-term memory, and social participation abilities.**

**
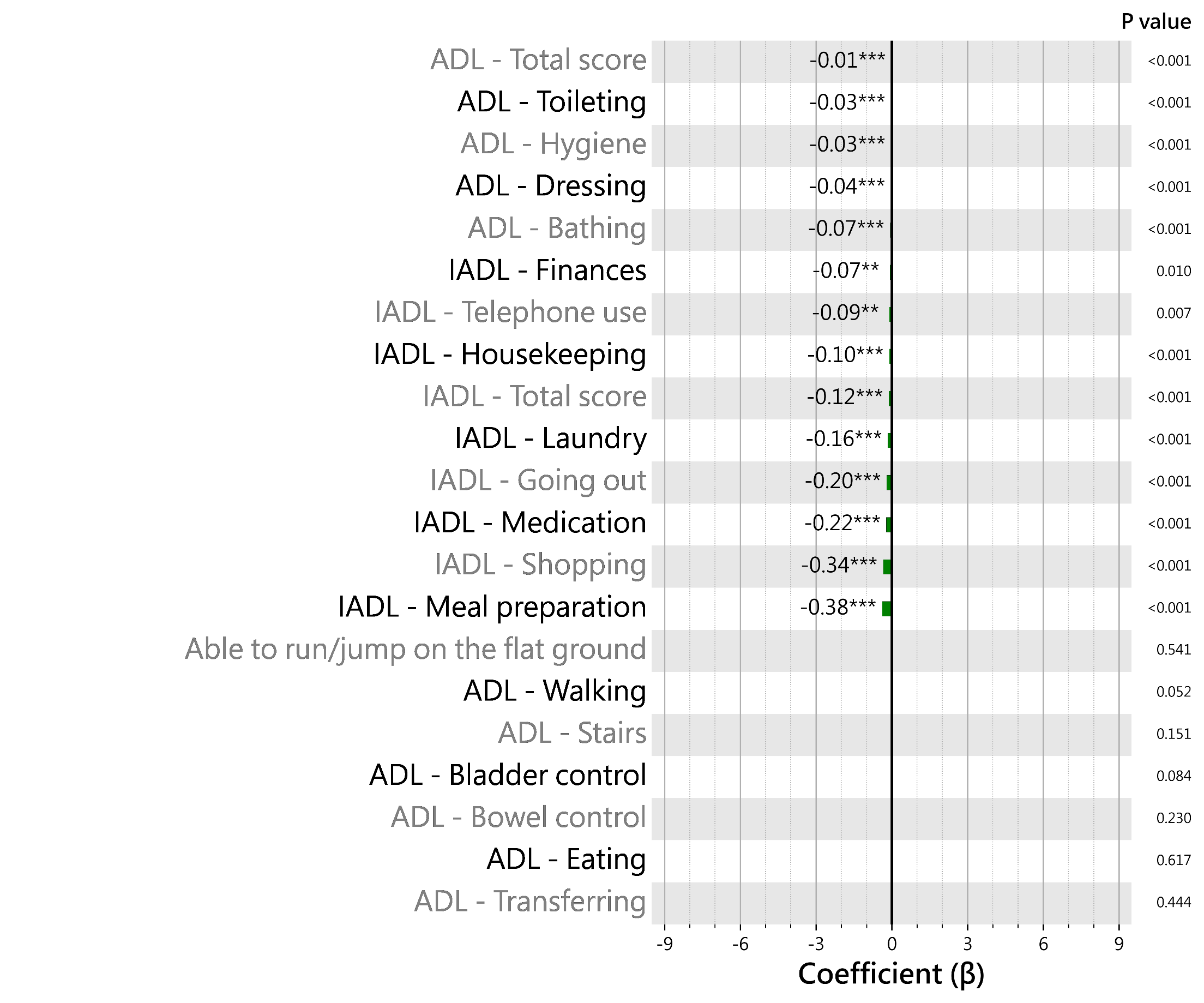
**

**Fig S8. MRA results- care recipient's ADLs and IADLs scale.**

**
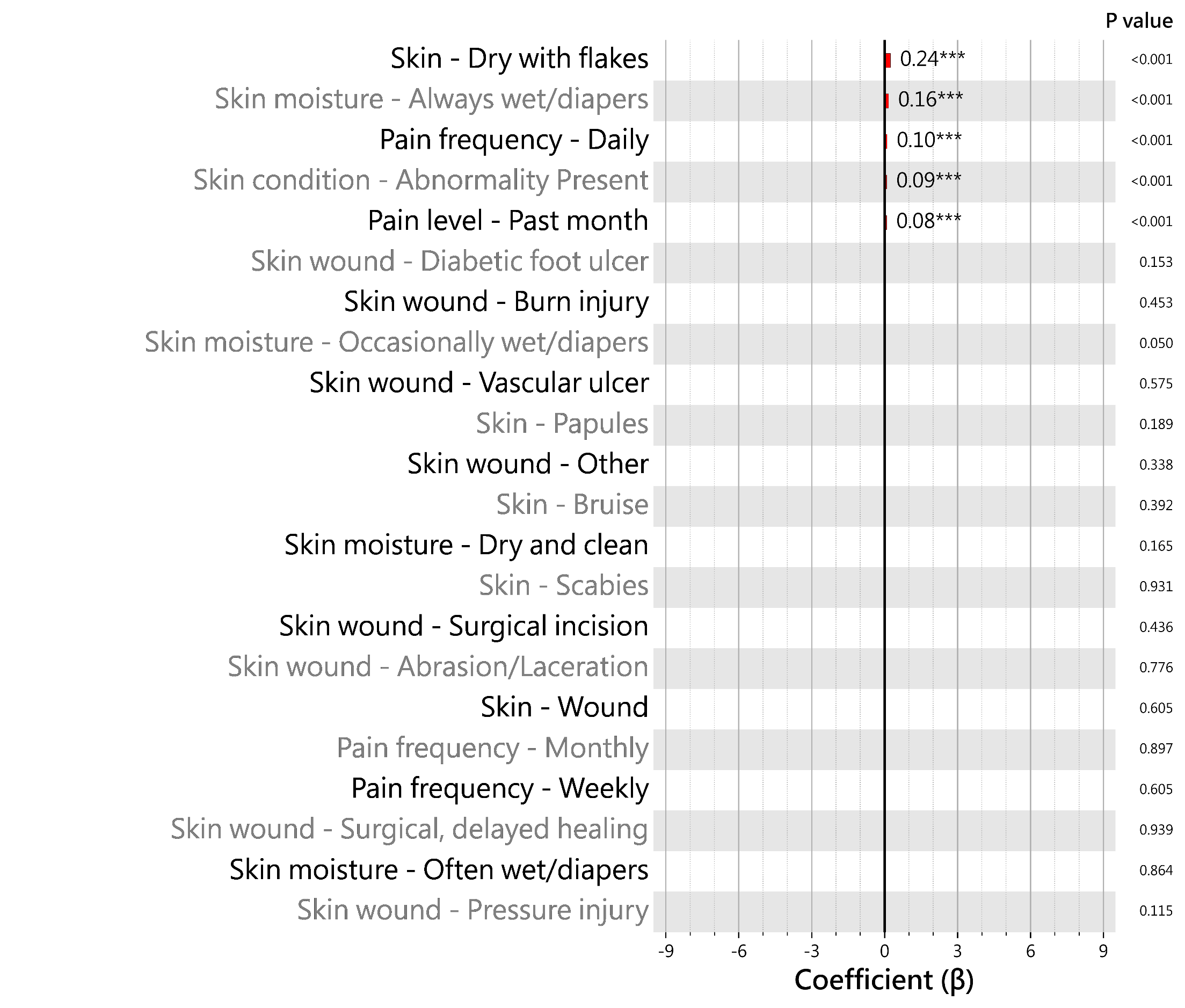
**

**Fig S9. MRA results- care recipient's pain, skin, and wound condition.**

**
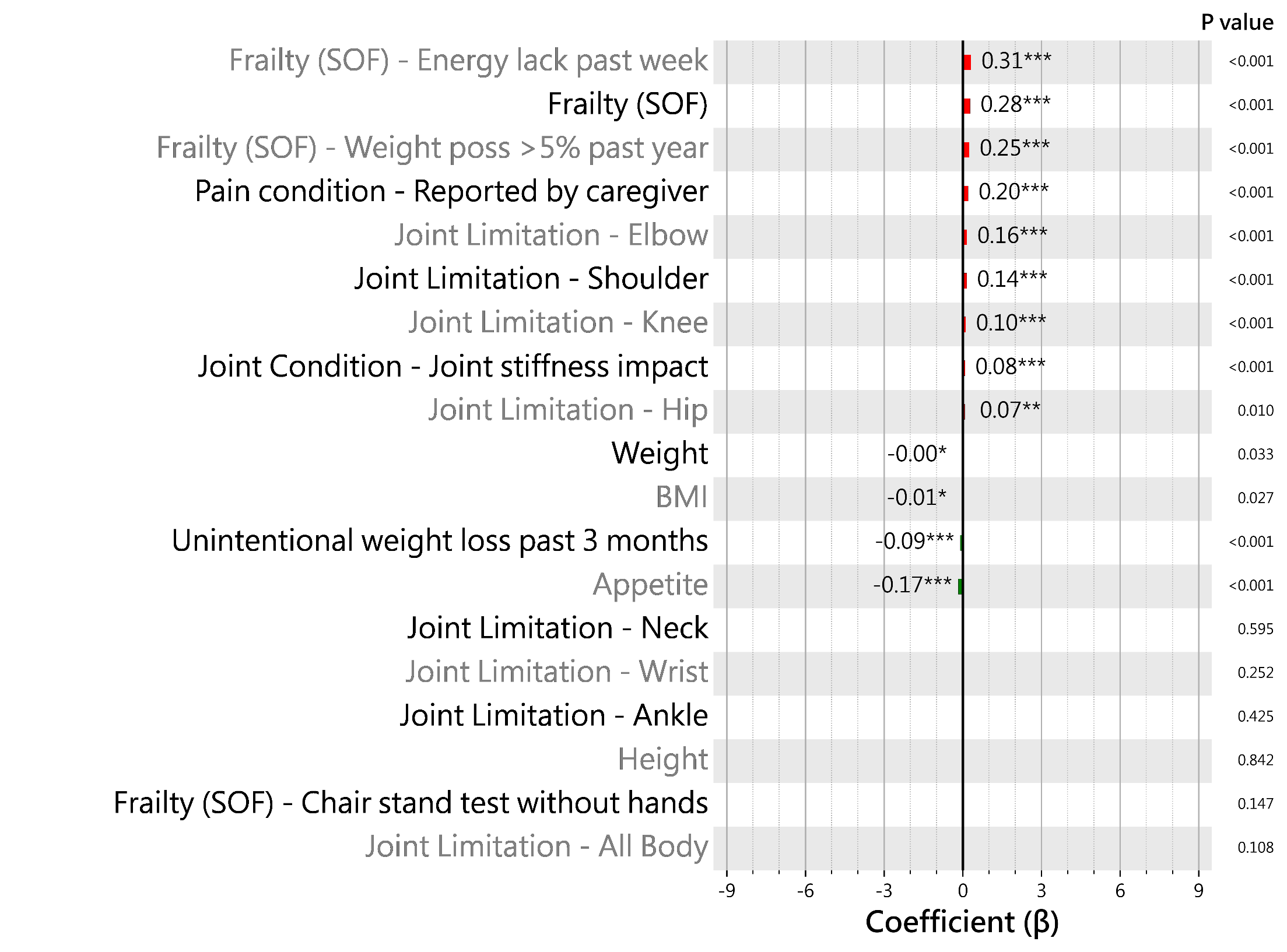
**

**Fig S10. MRA results- care recipient's joint, nutrition, and frailty (SOF) condition.**


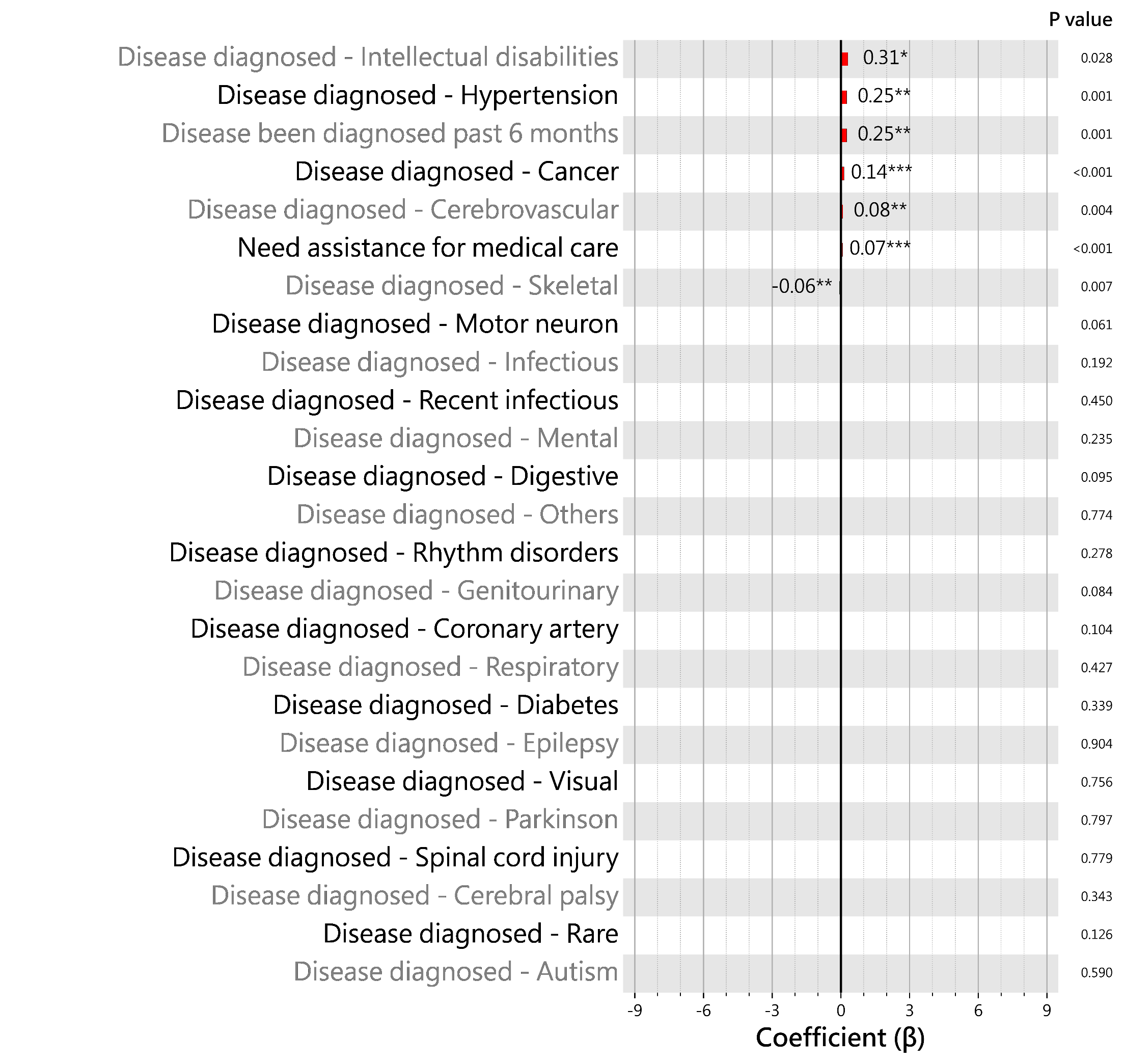


**Fig S11. MRA results- care recipient's medical history.**

**
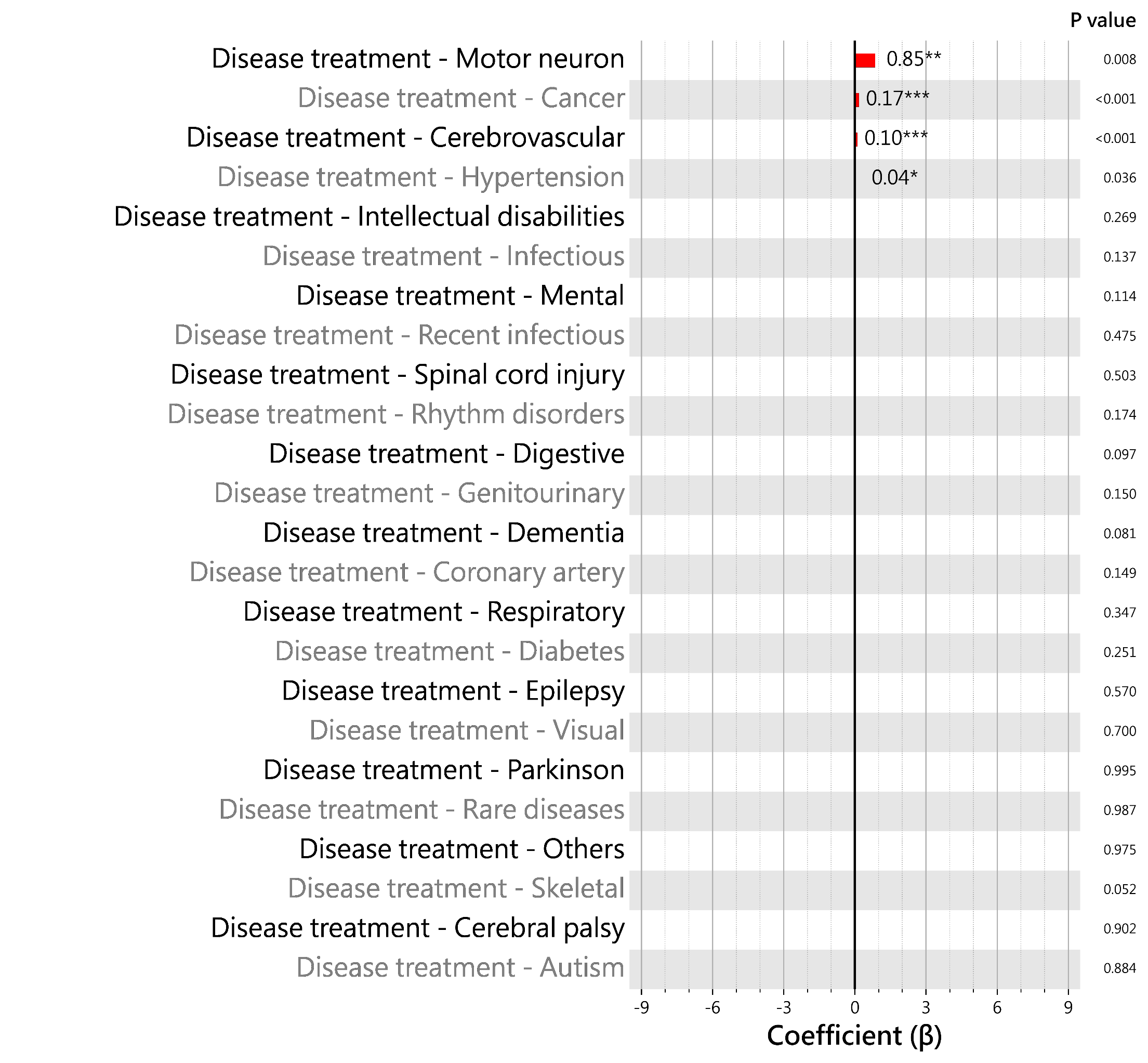
**

**Fig S12. MRA results- care recipient's medical history (treatment receiving).**

**
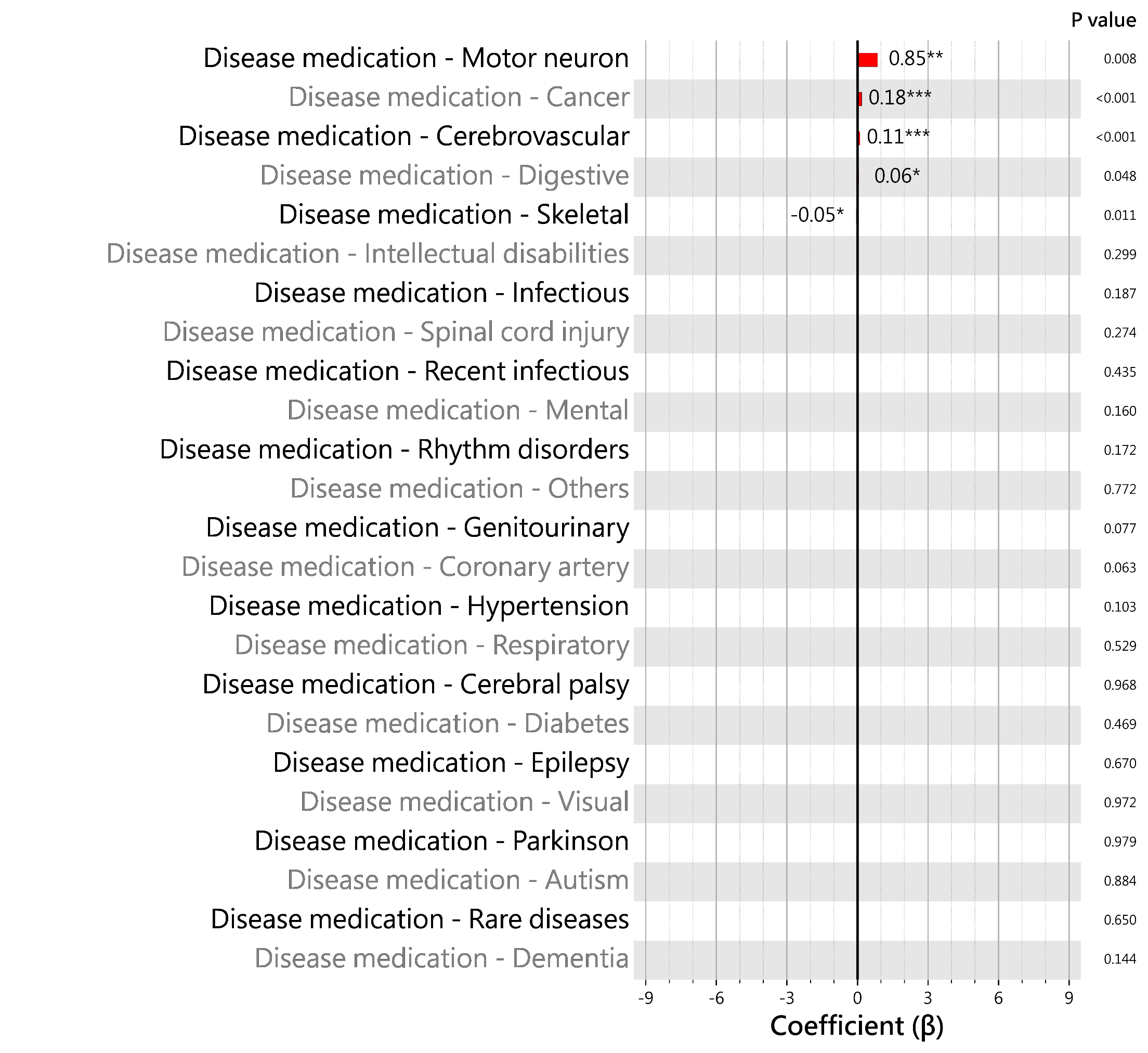
**

**Fig S13. MRA results- care recipient's medical history (medication taking).**

**
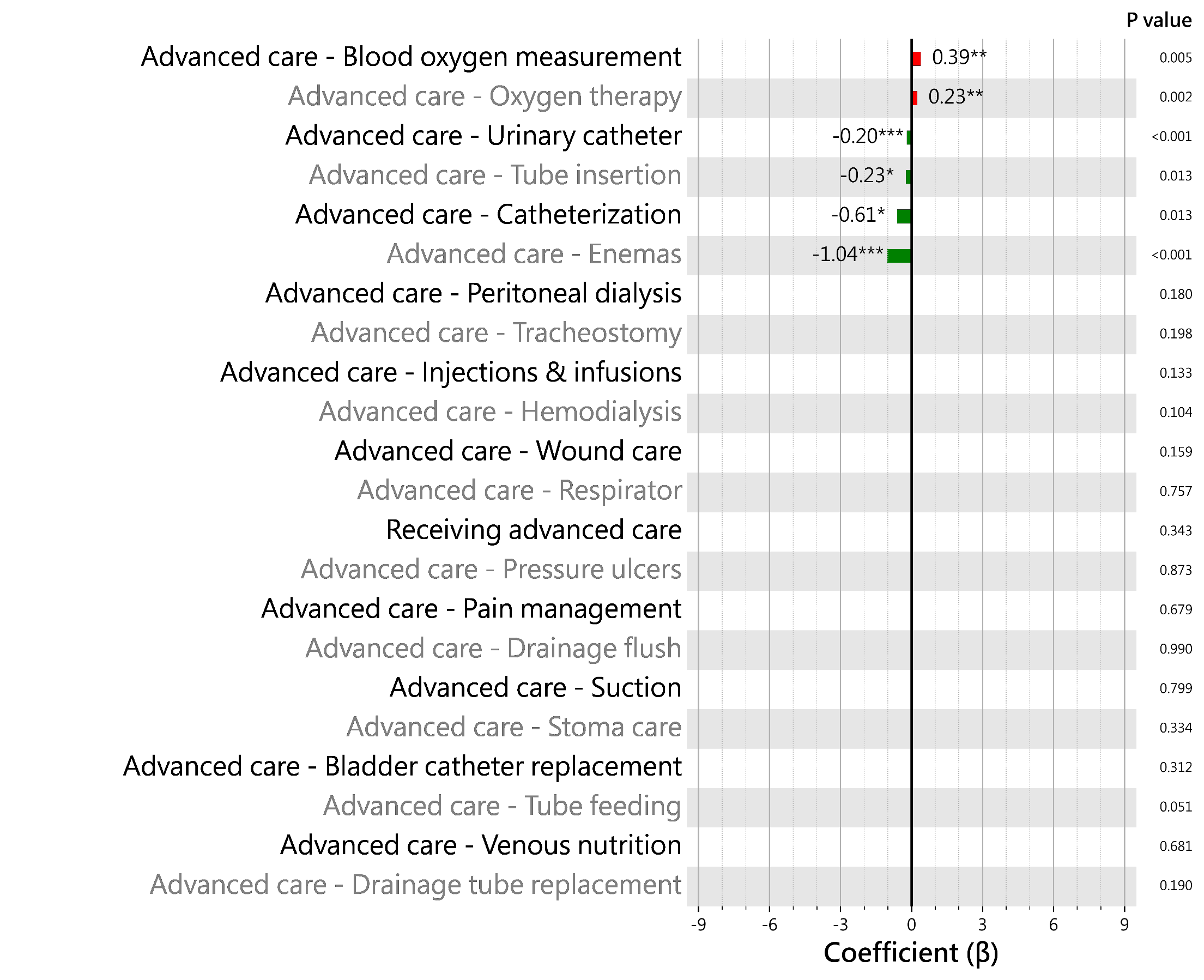
**

**Fig S14. MRA results- care recipient's advanced care status.**

**
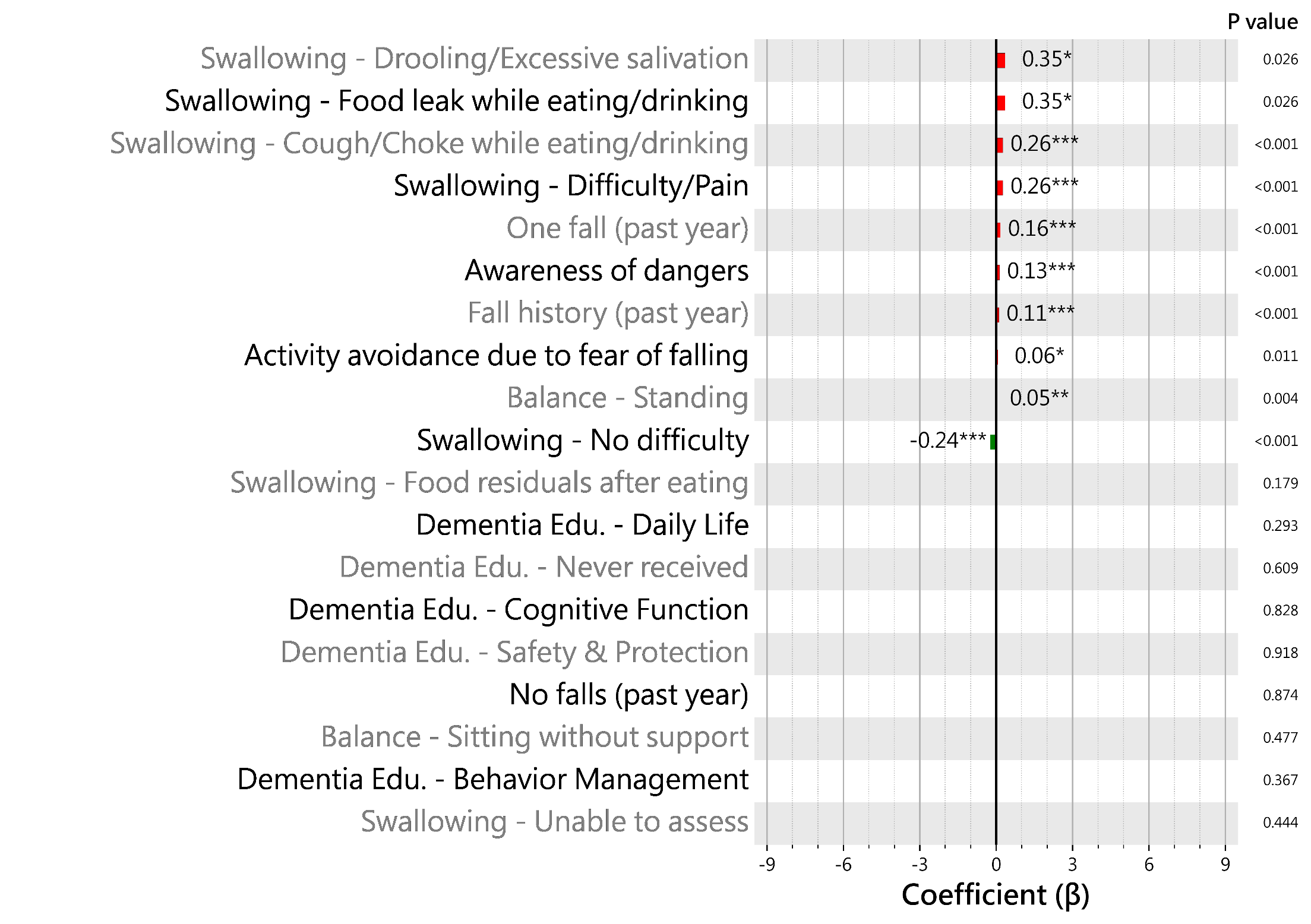
**

**Fig S15. MRA results- care recipient's swallow condition and fall risk.**

**
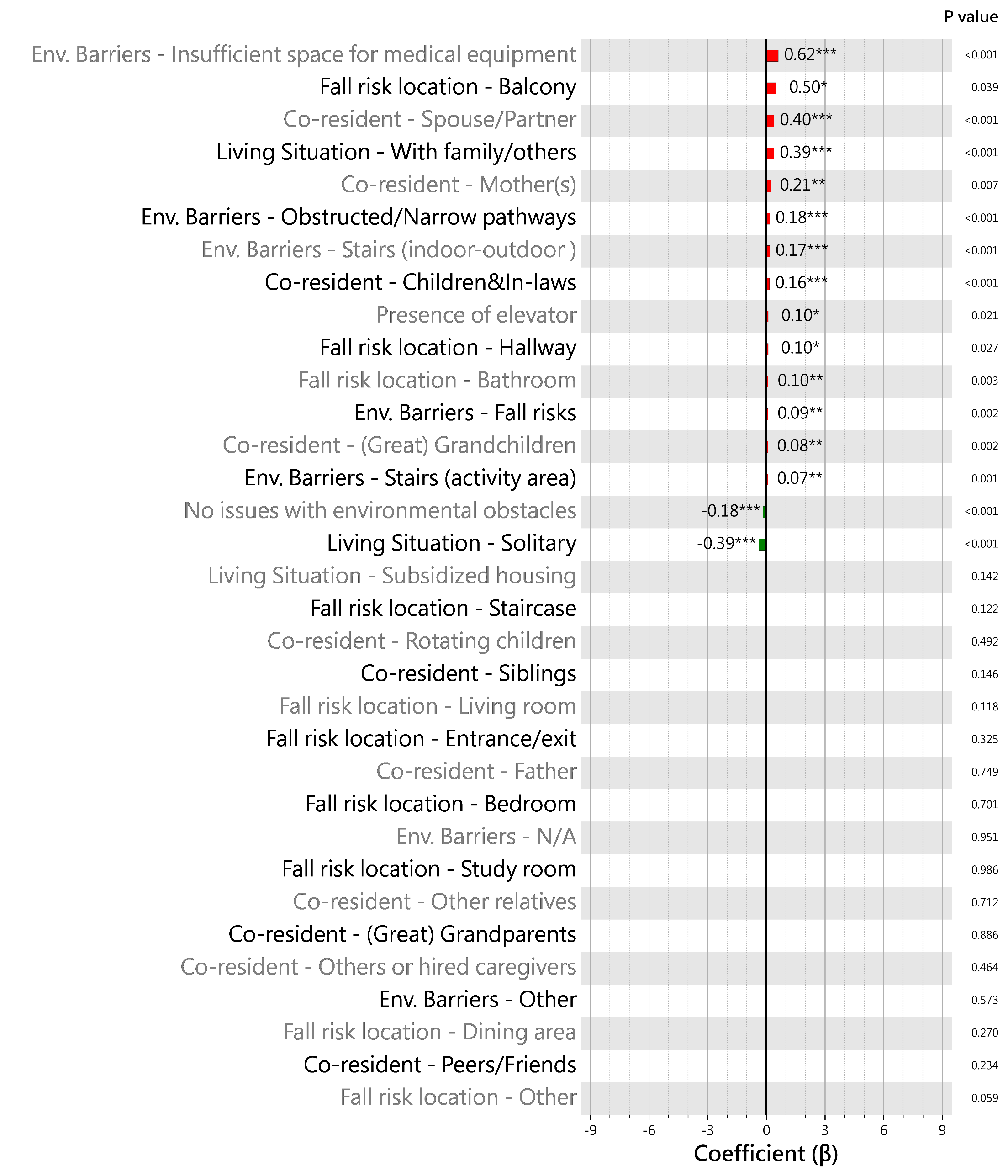
**

**Fig S16. MRA results- care recipient's co-resident and environment status.**


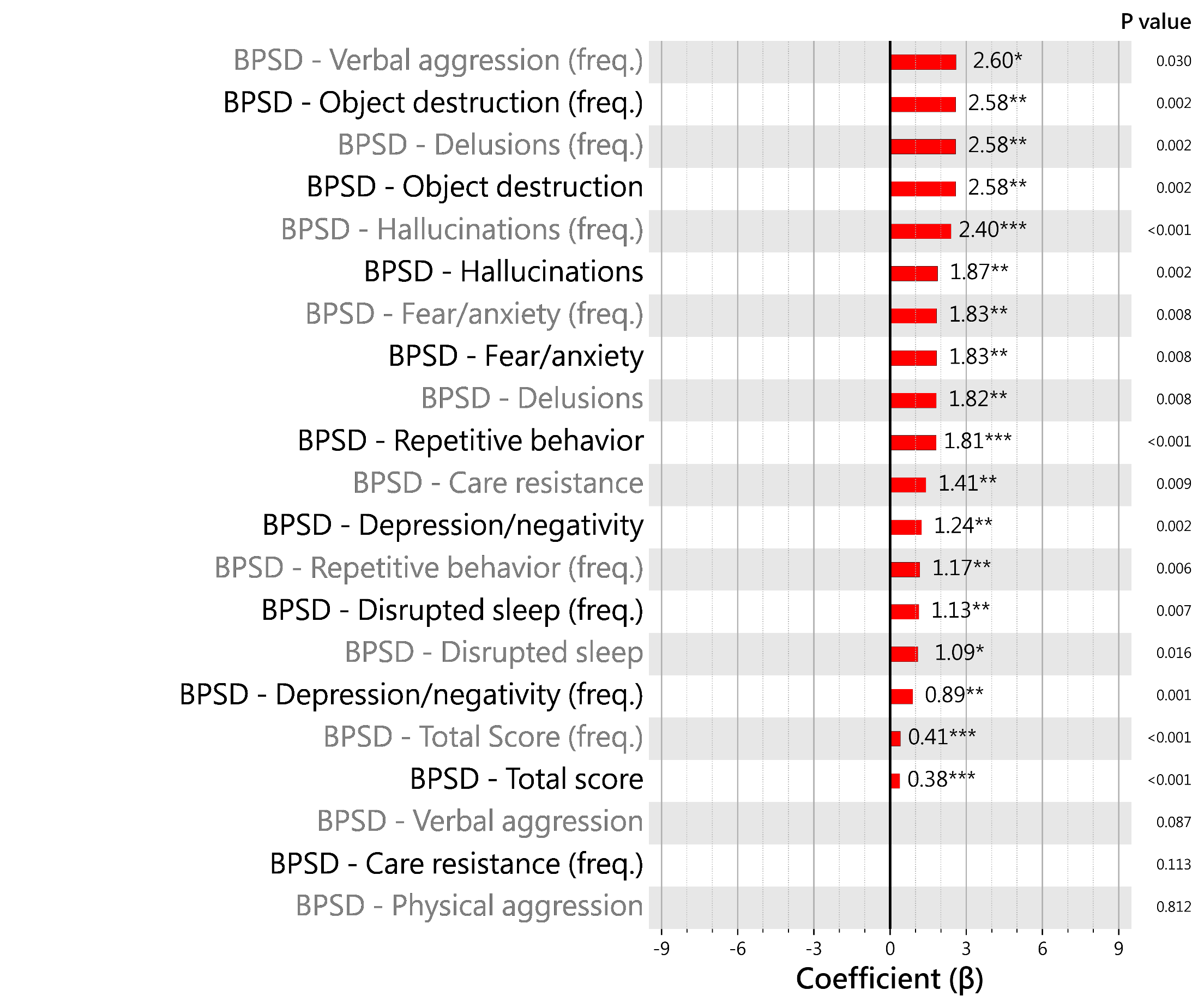


**Fig S17. MRA results- care recipient's BPSD condition.**

**
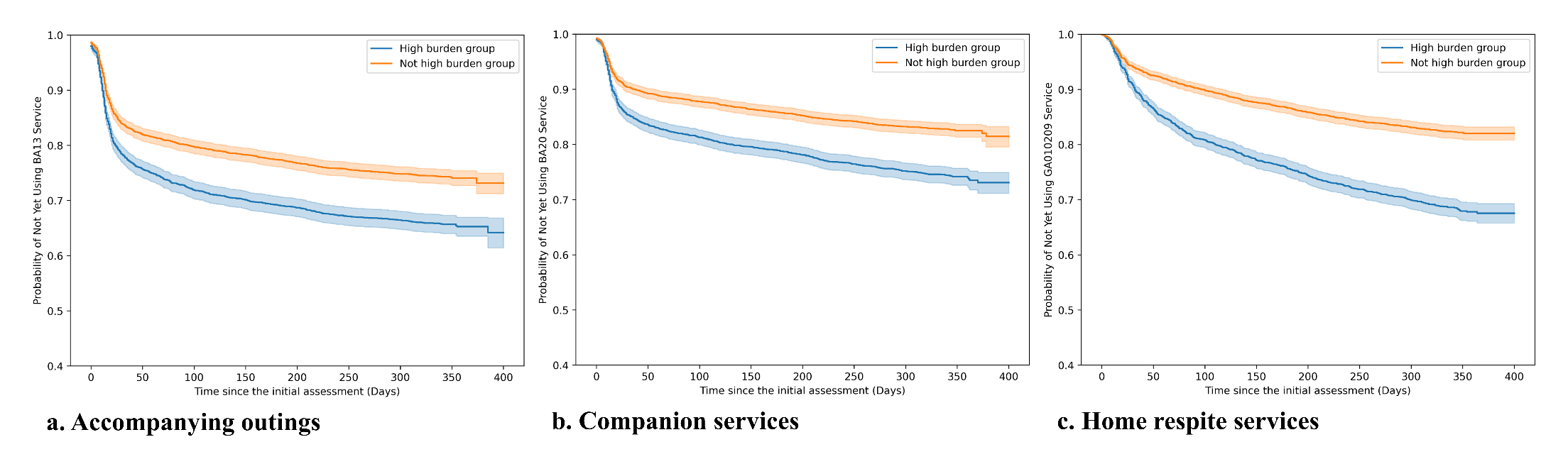
**

**Fig S18. Kaplan–Meier analysis: first adoption timing of LTC services (statistically significant).**
